# Quantitative super-resolution imaging of pathological aggregates reveals distinct toxicity profiles in different synucleinopathies

**DOI:** 10.1073/pnas.2205591119

**Published:** 2022-10-07

**Authors:** Michael J. Morten, Liina Sirvio, Huzefa Rupawala, Emma Mee Hayes, Aitor Franco, Carola Radulescu, Liming Ying, Samuel J. Barnes, Arturo Muga, Yu Ye

**Affiliations:** ^a^Department of Brain Sciences, Imperial College London, London W12 0NN, United Kingdom;; ^b^UK Dementia Research Institute at Imperial College London, London W12 0BZ, United Kingdom;; ^c^Instituto Biofisika, University of the Basque Country (Universidad del País Vasco/Euskal Herriko Unibertsitatea), Leioa, 48940 Spain;; ^d^National Heart and Lung Institute, Imperial College London, London W12 0BZ, United Kingdom

**Keywords:** super-solution imaging, protein aggregation, neurodegeneration, α-synuclein, proteasome

## Abstract

Protein aggregation is a hallmark of major neurodegenerative disorders. Increasing data suggest that smaller aggregates cause higher toxic response than filamentous aggregates (fibrils). However, the size of small aggregates has challenged their detection within biologically relevant environments. Here, we report approaches to quantitatively super-resolve aggregates in live cells and ex vivo brain tissues. We show that Amytracker 630 (AT630), a commercial aggregate-activated fluorophore, has outstanding photophysical properties that enable super-resolution imaging of α-synuclein, tau, and amyloid-β aggregates, achieving ∼4 nm precision. Applying AT630 to *App^NL-G-F^* mouse brain tissues or aggregates extracted from a Parkinson’s disease donor, we demonstrate excellent agreement with antibodies specific for amyloid-β or α-synuclein, respectively, confirming the specificity of AT630. Subsequently, we use AT630 to reveal a linear relationship between α-synuclein aggregate size and cellular toxicity and discovered that aggregates smaller than 450 ± 60 nm (aggregate_450nm_) readily penetrated the plasma membrane. We determine aggregate_450nm_ concentrations in six Parkinson’s disease and dementia with Lewy bodies donor samples and show that aggregates in different synucleinopathies demonstrate distinct potency in toxicity. We further show that cell-penetrating aggregates are surrounded by proteasomes, which assemble into foci to gradually process aggregates. Our results suggest that the plasma membrane effectively filters out fibrils but is vulnerable to penetration by aggregates of 450 ± 60 nm. Together, our findings present an exciting strategy to determine specificity of aggregate toxicity within heterogeneous samples. Our approach to quantitatively measure these toxic aggregates in biological environments opens possibilities to molecular examinations of disease mechanisms under physiological conditions.

## Significance

We have developed an approach to define the size of small aggregates involved in neurodegenerative disorders at ∼4 nm precision in live cells and in pathological model mouse tissues. We show that aggregates below 450 nm are harmful and readily penetrate cells. Once they invade cells, aggregates are surrounded by proteasomes, which are responsible for protein destruction, leading to aggregate removal. We further isolated aggregates from Parkinson’s disease and dementia with Lewy bodies donors, two distinct diseases that involve aggregates assembled from the same protein, α-synuclein, to validate that our 450 nm aggregate definition correlates with the measured toxicity level and reveal that different synucleinopathies have distinct toxicity profiles. Our method offers possibilities to strategize for individual disease prognosis.

Insoluble deposits of misfolded proteins that form aggregates are hallmarks of major neurodegenerative disorders such as Alzheimer’s disease (AD) and Parkinson’s disease (PD) (1–4). The molecular processes involved in aggregate assembly have been characterized for several misfolded proteins, including tau (5), amyloid-β (Αβ) (6), and α-synuclein (αS) (7). The occurrence of filamentous aggregates (fibrils) of tau and Αβ in AD, and αS fibrils in synucleinopathies such as PD and dementia with Lewy bodies (DLB), led many to examine a potential toxic and disease-causing nature of fibrils (e.g., refs. 7–9). More recently, a growing number of studies suggest that the smaller aggregates, including oligomeric aggregates (oligomers) (10–12), which are approximately spherical entities below 20 nm in diameter and assembled from a low number of misfolded proteins at the early stages of aggregation (13–15), are the most toxic aggregate species. These aggregates are potent in penetrating lipid membranes (16, 17), inducing oxidative stress (18), and activating proinflammatory signaling (19–21) and serve as “seeds” for further aggregation and spreading to other cells (22). In contrast to stable and elongated fibrils, small aggregates are usually soluble entities with largely globular structures whose features may be difficult to detect by conventional microscopy, therefore presenting a challenge to their structural characterization (23, 24). A second challenge arises from the limitation in strategies that are available to quantify different aggregate species in situ in order to test any putative relationship to cellular toxicity. Developing novel approaches to address these challenges would provide insight into the early stages of multiple neurodegenerative disorders.

Aggregation of misfolded proteins is a complex process both in vitro and in a physiological context, and the relative levels and sizes of small aggregates and fibrils change as aggregation progresses (25–28). Distinguishing small aggregates from fibrils based on their structure and quantifying their abundance is important, as different aggregates are thought to be processed through distinct cellular mechanisms. For example, we previously demonstrated that proteasomes disassemble fibrils into smaller toxic entities in vitro (29), whereas oligomers and other small aggregates carrying distinct ubiquitin modifications may be directly degraded by the proteasome (30). Combining fluorescence microscopy with fluorophores such as Thioflavin S and Thioflavin T (ThT) has enabled detection of aggregate structures in vitro and in biological samples (31, 32). ThT reversibly binds β-sheet structures in amyloid assemblies and increases its fluorescence intensity upon binding (33). Several super-resolution approaches were previously developed for ThT-based aggregate staining (34–36). Similar aggregate-activated fluorophores such as pFTAA, Nile Red, and Th-X have also been developed for aggregate detection using a range of dedicated fluorescence techniques (29, 37–39). These fluorophores may, however, preferentially detect stable fibrils, which contain extensive β-sheet structures, while oligomers are more difficult to characterize owing to the low fluorescence intensity expected from the staining of these smaller structures (40, 41).

It is possible to super-resolve aggregates that are assembled from misfolded proteins covalently labeled with Alexa-fluorophores (30, 42, 43). The stochastic switching between bright and dark states of organic fluorophores such as Alexa Fluor 647 (Alexa647) is excellent for single-molecule localization microscopy (SMLM) (44). However, Alexa labeling could interfere with protein aggregation of both tau and αS and, in some cases, prevent fibril formation (15, 30, 45). Recent applications of a technique called DNA-PAINT have enabled SMLM imaging of oligomers and small aggregate species in the range of 20 to 200 nm in diameter using aggregate-binding aptamers and antibodies (46, 47). Although not suitable for imaging live cells, oligomer sizes detected by DNA-PAINT are in agreement with those measured by atomic force microscopy (41, 48). However, these approaches require technical expertise to carefully execute the multiple procedures and require long imaging times. More recently, new commercial aggregate-activated fluorophores, such as ProteoStat and the Amytracker series (49, 50), have gained popularity as aggregate-specific stains (e.g., refs 51–55). Both fluorophores claim to offer increased sensitivity and specificity for aggregates compared to ThT.

In this study, we demonstrate that both ProteoStat and Amytracker 630 (AT630) are suitable for quantitative SMLM imaging of aggregates at ∼4 nm precision. AT630 staining, in particular, enabled sensitive and quantitative detection of aggregate species down to ∼10 nm in size in live cells and *ex vivo* brain tissue. We showed that aggregates found in live HEK293A cells or in fixed mouse brain sections could be super-resolved following AT630 staining. Critically, AT630 staining revealed that the plasma membrane effectively prevented aggregates over 450 ± 60 nm from entering the cell, and we quantitatively characterized aggregates that invaded the intracellular space. Using CRISPR-engineered cells in which endogenous proteasomes were tagged with enhanced green fluorescent protein (eGFP), we showed that internalized aggregates became surrounded by proteasomes, suggesting that proteostasis mechanisms respond promptly to proteotoxicity. Finally, we demonstrated that the size of the membrane-penetrating aggregates detected by AT630 correlated with cytotoxicity. We validated our observation and quantified aggregate_450nm_ toxicity using PD- and DLB-derived aggregates, showing that αS aggregates differ in potency in toxicity depending on the pathology they originated from. Our approach to detect different aggregate species through SMLM characterization provides a straightforward and reliable in situ approach to quantify the fraction of toxic aggregates.

## Results and Discussion

### Sensitive Aggregate Staining with ProteoStat and AT630.

**Fig. 1.**
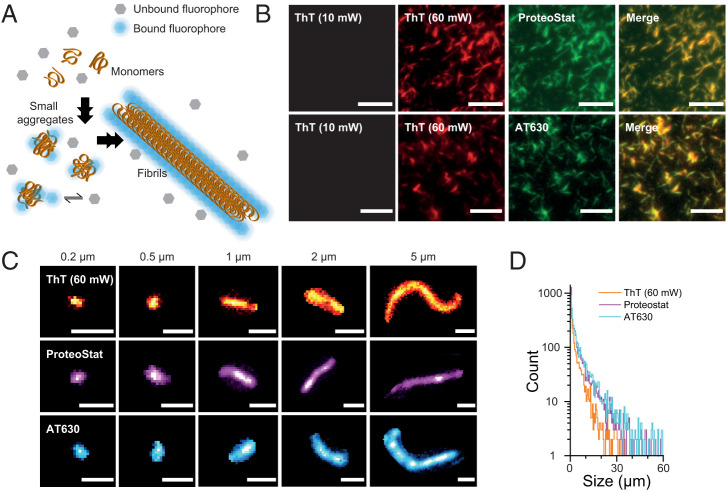
Detecting aggregate species with ThT, ProteoStat, and AT630. (*A*) Schematic representation of small aggregate and fibril detection with fluorophores that reversibly bind to aggregates. Unbound fluorophores (in gray) are nonfluorescent. Binding to aggregates locks the fluorophores in a high-fluorescence conformation (in blue), from which aggregate morphology can be detected. (*B*) Representative TIRF images of aggregates assembled from recombinant αS stained with ThT and ProteoStat (*Top*) or ThT and AT630 (*Bottom*). Laser power was set at 10 mW unless otherwise stated. Scale bars represent 5 µm. (*C*) TIRF images of typical aggregates ranging from 0.2 to 5 µm in size detected with ThT (*Top*), ProteoStat (*Middle*), and AT630 (*Bottom*). Scale bars represent 1 µm. (*D*) Semilog plot of frequency count of aggregates detected up to 60 µm with each fluorophore. Kolmogorov-Smirnov test indicates that AT630 and Proteostat detected a significantly greater number of aggregates (N_AT630_ = 4,417; N_ProteoStat_ = 4,473; N_ThT_ = 2,353) and are more faithful at detecting larger aggregates as evidenced by their greater medians and interquartile ranges (IQRs): IQR (median[IQR])_AT630_ = 2,428[655–7,260] nm; median[IQR]_ProteoStat_ = 1,763[452–5,712] nm; median[IQR]_ThT_ = 1,499[490–4,432] nm; *P* < 0.0005). See *SI Appendix*, Fig. S1 for biophysical properties of the three fluorophores.

We first set out to determine the specificity of ProteoStat and AT630 for different aggregate species and compared these with the widely used ThT (56–58). Since the excitation wavelength used and the emission maximum of ThT differs from those of ProteoStat and AT630 (*SI Appendix*, Fig. S1*A*), we were able to co-stain aggregates assembled from recombinant αS with ThT and ProteoStat or with ThT and AT630 (Fig. 1*A*). Assembled aggregates were imaged on a custom-built total-internal reflection fluorescence (TIRF) microscope, following our established protocols (29) (see *Materials and Methods*). We determined the photophysical properties for all three fluorophores and found that both ProteoStat and AT630 were brighter than ThT (*SI Appendix*, Fig. S1*B*), which required a sixfold increase in excitation laser power to reach an acceptable level of detection (Fig. 1*B*). Merging the same field of view staining of ProteoStat and ThT or AT630 and ThT showed complete overlap of aggregates in the two images, indicating that ProteoStat and AT630 both detected all aggregate species recognized by ThT (Fig. 1*B*). For controls, we showed that fluorescence emission in the absence of aggregates, in the presence of the aggregates but absence of fluorophores, or in the presence of monomeric αS and fluorophores was negligible (*SI Appendix*, Fig. S2). This indicates that both fluorophores have a high specificity for aggregate structures and high signal-to-noise ratios.

Our imaging approach detected a wide range of αS aggregate sizes by fluorophore staining (Fig. 1*C*). We previously defined aggregate size from TIRF imaging as the longest distance that could be measured within an aggregate entity, defining species <1 µm as “small aggregates” and “fibrils” for species >1 µm (29) (see *Materials and Methods*). Importantly, in addition to characterizing large fibril structures, small aggregates, whose molecular features reach below the ∼200 nm diffraction limit, were easily identified even at low laser power (10 mW unless stated otherwise). The abundance of aggregates detected with all three fluorophores is plotted by size (Fig. 1*D*). AT630 and ProteoStat were able to detect a significantly greater number of aggregates at the same aggregate concentration. We conclude that both ProteoStat and AT630 are more adept at uniformly staining aggregates than ThT (Fig. 1*D*) and require less laser power for reliable aggregate detection, thus reducing potential phototoxic damage to biological samples.

To account for the presence of internal structures and aggregate conformations that might not have been recognized by ThT in Fig. 1*B*, we further assembled aggregates from αS monomers covalently labeled with Alexa647. We developed a strategy mixing different fractions of Alexa647-labeled and unlabeled αS to overcome potential steric interference that Alexa647 may have on αS aggregation, which could prevent assembly of fibrils (30). We optimized this fraction (10% Alexa647-labeled αS) to allow formation of fibrils that incorporated Alexa647-labeled αS (*SI Appendix*, Fig. S3*A*). Fibril structures were reconstructed in three dimensions (3D) using an astigmatism SMLM imaging method (59), which confirmed that labeling was largely uniform over the fibril structures (*SI Appendix*, Fig. S3*B* and Video S1; technical details further below). Our SMLM approach achieved 21 ± 1 nm planar and 84 ± 2 nm axial resolution (see *Materials and Methods* and *SI Appendix*, Fig. S4). ProteoStat and AT630 both stained Alexa647-labeled αS aggregates, and the detected features matched with the fluorescence detected from Alexa647 (*SI Appendix*, Fig. S3*C*). These results confirm that ProteoStat and AT630 detected all aggregate species present in the sample.

Imaging individual aggregates allowed us to explore the relationship between the total fluorescence intensity detected from each aggregate versus its size (*SI Appendix*, Fig. S3*D*). The total fluorescence intensity detected from each aggregate can be assumed as a product of the fluorophore brightness and the number of fluorophores bound to each aggregate. Both ProteoStat and AT630 demonstrated greater fluorescence intensities with aggregate size compared to ThT and Alexa647 labeling in *SI Appendix*, Fig. S3*D*. Since the brightness of ProteoStat and AT630 is greater than that of ThT but not as bright as that of Alexa647 (*SI Appendix*, Fig. S1*B*), we would therefore expect that the increased total intensities of ProteoStat and AT630 originate from higher densities of fluorophores bound to each aggregate. The increased density of fluorophores reflects an expected higher density of aggregate structures than the 10% of monomers that were labeled with Alexa647 (29). Overall, these data suggest that AT630 and ProteoStat more sensitively detected amyloid structures than Alexa647 and ThT. Furthermore, these data highlight the advantages of aggregate-activated fluorophores over the Alexa647-conjugation approach.

### Super-resolution of Aggregates with ProteoStat and AT630 at ∼4 nm Precision.

**Fig. 2.**
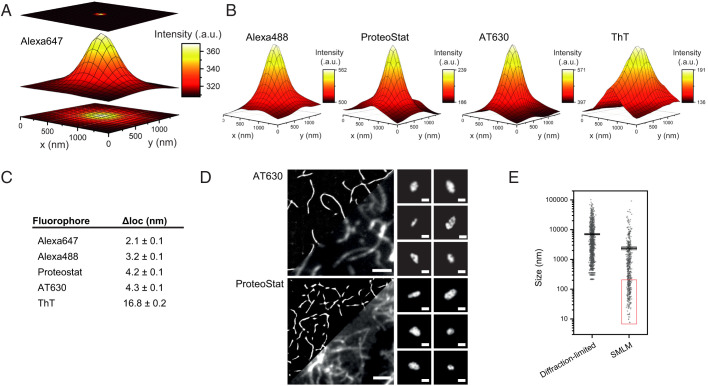
Super-resolution of aggregates by SMLM with ProteoStat and AT630. (*A*) The principle of PSF analysis. Emission from Alexa647 is detected as a diffraction-limited spot in 2D (*Bottom*). The emission may be presented in 3D using fluorescence intensities registered at each pixel (*Middle*). The PSF is mathematically modeled to pinpoint a more precise location of the fluorophore (*Top*). (*B*) The average PSFs detected from SMLM imaging of aggregates using Alexa488, ProteoStat, AT630, and ThT. (*C*) Table of values for measured localization precision (Δloc) calculated from the PSF of each fluorophore using $\Delta \mathrm{loc}=\mathrm{FWHM}/\sqrt{\mathrm{photons}}$. (*D*) (*Left*) Comparison of recombinant AT630- and ProteoStat-stained αS aggregates imaged by SMLM reconstruction (see also Videos S3 and S4) and conventional diffraction-limited TIRF methods. Scale bars represent 2 μm. (*Right*) Representative images of smaller aggregates detected using SMLM. Scale bars represent 100 nm. (*E*) An example of improvement in determining distribution of aggregates’ size as measured from diffraction-limited or super-resolution images (in the red rectangle) using AT630 (*n* = 1,273 for the diffraction limited and *n* = 576 for the SMLM samples). a.u., arbitrary units.

Accurately determining the size of aggregates below ∼200 nm is challenging due to the Abbe diffraction limit. We therefore used SMLM to characterize the structural features of aggregates in finer detail. We initially imaged aggregates in two dimensions (2D) (*SI Appendix*, Fig. S5) and in 3D (*SI Appendix*, Fig. S3*B*) using fibrils labeled with Alexa647 or Alexa488 (44). We used high laser powers and an imaging buffer containing a reducing agent and an oxygen scavenger system (see *Materials and Methods*) to induce the fluorophores to stochastically switch on and off, allowing densely packed single molecules to be distinguished temporally (60–62). The point-spread-functions (PSFs) measured from the emission of individual fluorophores were fitted to Gaussian profiles, and these were used to calculate the position of each fluorophore with greater accuracy (62). Fig. 2*A* shows the average PSF profile of Alexa647 conjugated on αS aggregates collected for SMLM. The localization precision (Δloc) was determined from the number of photons detected and the full width half maximum (FWHM) of the PSF profile of Alexa647 (see *Materials and Methods*). Similarly, average PSFs were used to calculate the localization precision of Alexa488, ProteoStat, AT630, and ThT (Fig. 2*B* and Videos S2–S4). Alexa647 and Alexa488 achieved the highest localization precisions at 2.1 ± 0.1 nm and 3.8 ± 0.1 nm, respectively (Fig. 2*C*). The average PSF profiles of AT630 and ProteoStat also gave excellent signal-to-noise profiles and achieved localization precisions (4.2 ± 0.1 nm and 4.3 ± 0.1 nm, respectively) in range with the Alexa fluorophores. As expected, the localization precision determined for ThT was lower, achieving 16.8 ± 0.2 nm, similar to previously published values (57).

ProteoStat and AT630 both enabled SMLM imaging of αS aggregates assembled in vitro and revealed structural details of aggregates from >10 µm down to 10 nm in size (Fig. 2 *D* and *E* and Videos S3 and S4). We defined the size of each aggregate by thinning the aggregate shape to a path 1 pixel in width and calculated the aggregate size as the length of this 1-pixel-wide path (see *Materials and Methods*). From the reconstructed SMLM images, we found that the smaller aggregates within a few hundred nanometers in size were heterogeneous in structure; some appeared globular, while others were elongated and with apparent differences in ellipticity (Fig. 2*D*), similar to those reported by others (38, 56). Similar observations were made using the same staining approach to super-resolve aggregates assembled from tau or Aβ, demonstrating that sensitivity of detection was not dependent on the misfolded protein involved (*SI Appendix*, Fig. S6). These results together indicate that ProteoStat and AT630 enabled SMLM imaging of molecular features of aggregates at ∼4 nm localization precision, resolving the conformational heterogeneity in aggregate samples down to the same order of magnitude as the organic fluorophores.

### Aggregate Species below 450 ± 60 nm Readily Penetrate Plasma Membranes and Invade HEK293A Cells.

**Fig. 3.**
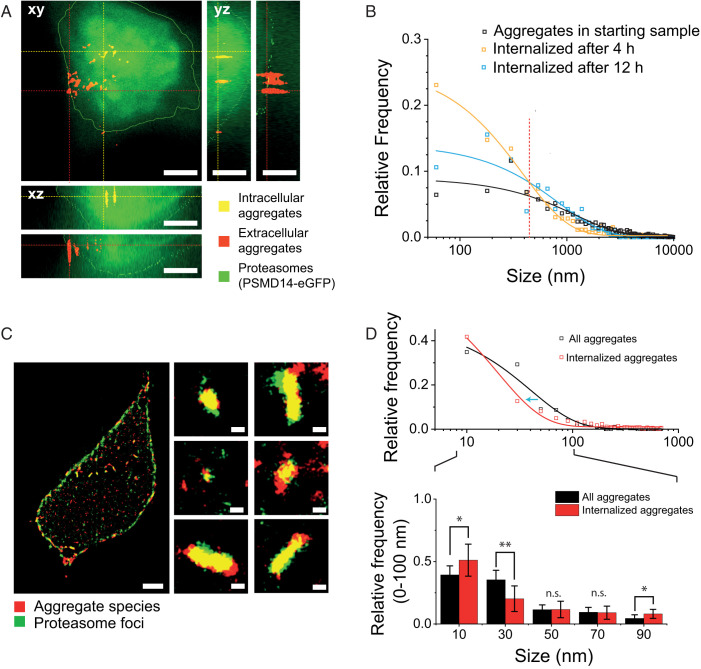
Imaging cell-penetrating aggregate species with size less than 450 ± 60 nm. (*A*) αS aggregates at 1 µM final concentration were added to HEK293A cells expressing proteasome subunit PSMD14-eGFP from the genomic loci (green). Aggregates were stained with AT630 (red). A typical cell is shown with profiles along the z axis and aggregates detected within the cell interior (merged colors show aggregates in yellow) or sitting on the external side of plasma membrane (aggregates appear red in the merged colors image) as indicated. Scale bars represent 5 µm. (*B*) Quantification of aggregates detected in live cells (N_4h_ = 14 cells; N_12h_ = 13 cells) within the intracellular domain after 4 h or 12 h (orange and blue, respectively) after incubation. The red dashed line indicates the intersection of the 4 h and 12 h curves at 450 ± 60 nm. Aggregate size distribution of the starting sample at time 0 h is shown in black (N_4h_ = 529; N_12h_ = 585; N_0h_ = 2,734). (*C*) Live super-resolution imaging by SMLM of AT630-stained aggregates (red) in the intracellular domain or blocked by the plasma membrane is shown. Proteasome foci (green) are represented by bright green spots. Typical foci-aggregate colocalizations are shown in the zoomed-in images. Scale bars represent 2 μm and 200 nm for the whole cell and insets, respectively (see also Video S5). (*D*) Quantification of aggregates detected by SMLM, showing the relative frequencies of aggregates with sizes between 0 and 100 nm. The top plot represents frequency distribution of aggregate sizes before and after they enter a cell. The blue arrow indicates the decrease in size of aggregates after entering a cell. The bottom bar chart focuses on aggregates smaller than 100 nm, and reports the mean aggregate size. Aggregates from 19 cells were imaged, with 893 total number of aggregates. Sonicated aggregates were imaged from five samples, with a total of 849 aggregates. A two-sample *t* test was used to calculate statistical significance between the groups of sizes, with n.s. (no significance) *P* > 0.05, *P < 0.05, and **P < 0.005. Error bars represent SD of triplicate measurements (n = 3).

To test whether the new fluorophores detect aggregates within a cellular environment, we incubated recombinant αS aggregates with HEK293A cells. αS aggregates released into the extracellular domain are known to be taken up by cells (reviewed in ref. 63). Here, we used a CRISPR-engineered line in which the genomic loci coding for a proteasomal subunit, *PSMD14*, was knocked in with an eGFP-coding sequence introduced to the 3′- end in frame with the gene, thereby fluorescently tagging endogenous proteasomes (64). Although suitable for SMLM (*SI Appendix*, Fig. S6), ProteoStat requires sample preparation through cell fixation and permeabilization steps prior to staining of intracellular aggregate species (see ref. 65 and manufacturer’s instructions); we therefore used AT630 to stain aggregates in live cells. We optimized staining time and washing procedure for AT630 to achieve the highest signal-to-background ratio for imaging, and we adjusted the TIRF microscope to apply a highly inclined and laminated optical sheet (HILO) imaging approach (66), which enabled axial illumination in the z direction throughout the whole cell volume (see *Materials and Methods*). The boundaries of the cell were defined from z-stacks of images taken 100 nm apart throughout the cell volume. Since proteasomes are dispersed throughout the cell interior (64, 67), we applied a rolling ball filter to the eGFP emission to reconstruct the cell volume and validated this approach comparing cell volumes detected from eGFP emission and a CellMask Plasma Membrane Stain (*SI Appendix*, Fig. S7). From the eGFP emission, we could distinguish between aggregates that had entered the cell from those that lay on the apparent apical side of plasma membrane (Fig. 3*A*). After 4 h incubation, we detected mostly smaller aggregates submicrometer in size (in yellow, Fig. 3*A*) within the intracellular domain, while the larger fibrils remained docked to the plasma membrane (in red, Fig. 3*A*). The relative frequency of internalized species was plotted by size, showing that the distribution of these aggregates was best described by an exponential function (orange curve, Fig. 3*B*). We subsequently compared this distribution to the internalized species after 12 h, in which we observed a greater frequency of larger aggregate species entering the cell (blue curve, Fig. 3*B*). As a comparison, we plotted the function based on the total number of aggregates detected in the starting sample (i.e., sample added at 0 h; black curve, Fig. 3*B*). The starting sample showed a lower relative prevalence of smaller aggregates than in the functions for both 4 and 12 h, suggesting a higher efficiency of these smaller aggregate species at crossing the plasma membrane. Given that the relative frequency of internalized aggregates at 4 and 12 h showed different distributions, the intersection at 450 ± 60 nm between the two curves represents the aggregate species that does not change in relative abundance over time (dashed line, Fig. 3*B*). Following this reasoning, we interpreted the crossover as a threshold to define a population of aggregates with sizes shorter than 450 ± 60 nm (aggregate_450nm_) that is able to traverse the cell membrane with increased efficiency.

Sonication has been shown to break apart fibrils and increase the abundance of smaller and more toxic aggregates in the mixture (29, 68); therefore, we repeated the aggregate invasion experiment with sonicated fibrils and followed the fate of aggregate_450nm_ after incubation for 24 h. We observed that the dispersed proteasomes assembled into foci structures (Fig. 3*C* and Video S5) and that areas of increased proteasome concentration condensed around internalized aggregates. Similar colocalizations of proteasomes concentrated around αS aggregates and in Lewy bodies is known (69–71), and the proteasome foci we observed here may be similar to stress-induced proteasome foci in previous reports (64, 67, 72, 73). Staining these aggregate-treated cells with AT630 followed by SMLM imaging (in Fluorobrite rather than SMLM imaging buffer, which is toxic to cells) revealed that many proteasome foci indeed colocalized with internalized aggregates. The AT630 staining performed in live cells was specific, as no meaningful fluorescence was detected in the absence of aggregates (*SI Appendix*, Fig. S8).

We subsequently determined the size of internalized aggregates by SMLM and compared their size distribution with the aggregates before they were deposited on the cells (Fig. 3*D*). A left shift in the distribution of intracellular aggregate size by 21 ± 3 nm was detected (blue arrow showing shift from black to red curve, Fig. 3*D*), indicating that internalized aggregates became smaller than before they were deposited on the cells. Focusing on aggregates with sizes <100 nm (inset, Fig. 3*D*), we found by using a two-sample *t* test that unlike the larger size groups, the smallest size group (10 nm) showed a marked increase in relative frequency once internalized. This is in line with the left shift of the red curve in Fig. 3*D* and suggestive of gradual disassembly/degradation of larger aggregates into the smaller species once internalized, similar to previous in vitro observations (29). Additional mechanisms may be involved in removing the smallest size group, perhaps similar to the ubiquitin-dependent mechanisms we showed before (30). Together, our data indicate that proteasomes target internalized aggregates, and the reduction in aggregate size in cells after 24 h suggests the observed foci contain active proteasomes. These results provide evidence for aggregates to promptly attract proteasomes, thus increasing local degradation activity to facilitate aggregate removal.

We recently proposed a putative cellular ability to reversibly increase local proteasome concentration and thereby degradation activity through assembly of “transient aggregate-associated droplets (TAADs)” (74). The observed colocalization between aggregates and proteasome foci is largely in agreement with the description of TAADs. Given that we typically observed aggregates inside cells after 4 h, we further suggest that the plasma membrane is readily permeable to smaller aggregates and filters out aggregates and fibrils above 450 ± 60 nm in size. Given the uncertainty range, it is likely that in reality this threshold represents a critical range of aggregate sizes, and aggregates that are significantly larger than this range are less likely to be internalized. The plasma membrane therefore serves as an effective protectant against larger fibrils (>450 ± 60 nm) while remaining vulnerable to small aggregates. It is tempting to speculate whether the reverse effect is also true—the plasma membrane may effectively contain the larger fibrils, while small aggregates escape into the extracellular domain, if below 450 ± 60 nm, and spread to adjacent cells.

### αS Aggregates below 450 ± 60 nm Correlate with Cytotoxicity.

**Fig. 4.**
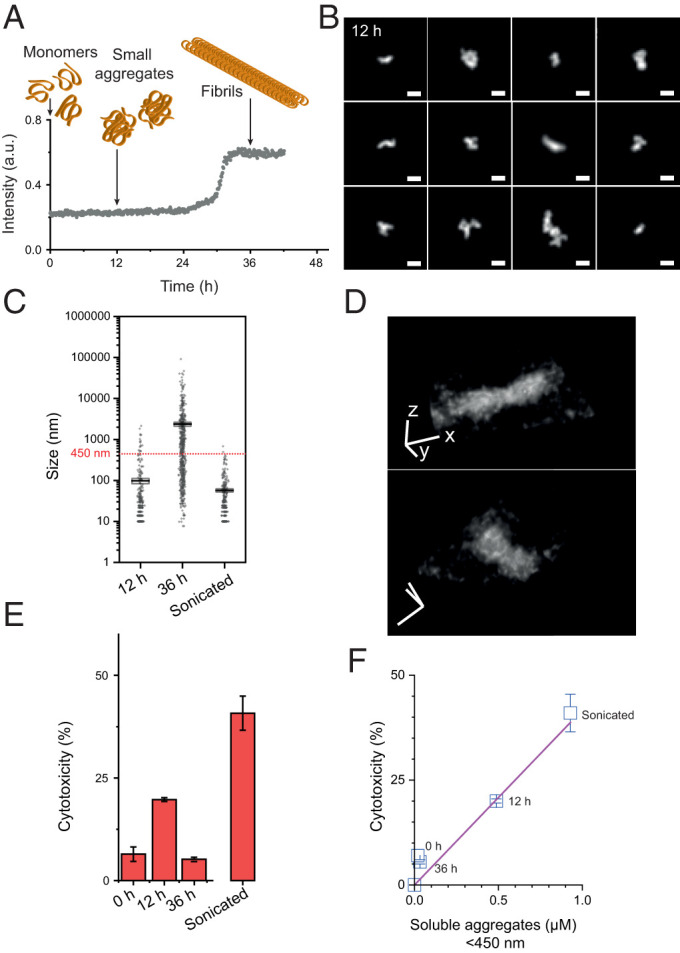
Quantitative super-resolution imaging correlates aggregate_450nm_ with toxicity. (*A*) The aggregation process of recombinant αS over time as measured by ThT. Arrows below cartoons represent the aggregate species (not drawn to scale) detected by TIRF imaging of aliquots taken at indicated time points (see *SI Appendix*, Fig. S9). (*B*) Representative small aggregates detected using AT630 at 12 h time point after aggregation. Scale bars represent 100 nm. (*C*) Quantification of aggregate species by size as detected by super-resolution for time points at 12 and 36 h and sonicated aggregates (N_12h_ = 313; N_36h_ = 595; N_son_ = 287). The horizontal dashed line shows the 450 nm size threshold calculated in Fig. 3*B*. (*D*) A typical AT630-stained αS aggregate reconstructed in 3D from super-resolution imaging, x-y resolution 24 nm, and z resolution 48 nm (see also Video S6). Scale bars represent 500 nm in x, y, and z directions. (*E*) Quantification of αS aggregate cytotoxicity as determined by LDH assay for aliquots taken and the time points indicated in *A*. Cells were treated with a final concentration of 1 μM αS. (*F*) Cytotoxicity-to-aggregate_450nm_ normalized to aggregate concentration shows a linear relationship. a.u., arbitrary units. Error bars represent SD of triplicate measurements (n = 3).

To determine whether the invasive aggregate_450nm_ we have identified in Fig. 3 may cause cytotoxicity (47), we set up a controlled αS aggregation reaction in vitro. The aggregation process of recombinant αS has been characterized by us and others (23, 30, 43), and we used these studies to guide the selection of time points at which aliquots should be taken (Fig. 4*A*). We expected no aggregates at time 0 h, when only monomers should be present, and mostly small aggregates 12 h after aggregation was initiated, before we observed any significant increase in ThT signal. A larger proportion of fibrils was expected at 36 h after the aggregation reaction had entered the stationary stage. Indeed, a predominant population of smaller aggregate species was detected after 12 h using AT630, no aggregates were observed at 0 h, and large numbers of fibrils were found at 36 h (Fig. 4 *B* and *C* and *SI Appendix*, Fig. S9 *A*–*C*). Again, we sonicated a sample of fibrils from the 36 h time point to generate a large population of smaller aggregates similar in size to those formed after 12 h (*SI Appendix*, Fig. S9*D*). The detected smaller aggregate species had distinctive structures, which we super-resolved in 2D and 3D (Fig. 4*D* and Video S6). Next, we treated HEK293A cells with a calculated equivalent αS monomer concentration of 1 µM taken at the different time points and measured the cytotoxicity using a lactate dehydrogenase (LDH) assay (Fig. 4*E*), which offers sensitive readouts of membrane permeabilization and LDH release (29). In agreement with our hypothesis, the aggregate sample at the 12 h time point was most harmful to cells, with 20.2 ± 0.6% cytotoxicity value of the positive control for the assay (lysis buffer treatment of cells). The monomer- and fibril-rich samples at 0 h and 36 h, respectively, induced smaller cytotoxic responses (5.5 ± 0.7% and 7 ± 2%, respectively) in comparison. As expected, sonicated fibrils from the 36 h time point resulted in significant cell death (41 ± 5% cytotoxicity; Fig. 4*E*). It is possible there are structural differences between αS aggregates from the 12-h time point and sonicated 36 h, such as those observed with tau where sonication increased epitope exposure (75). However, our results show that the size of the aggregates plays a prominent role when inducing cytotoxicity.

We subsequently sought to quantify the concentration of αS proteins that constitute the aggregate_450nm_ population at each time point. SMLM was used to determine aggregate size, and the fraction of aggregate_450nm_ was calculated from the total size of all aggregates in the sample compared to the sum of total sizes detected from aggregate_450nm_. Plotting the calculated concentrations of αS in aggregate_450nm_ versus the measured cytotoxicity of the different samples allowed us to fit a linear relationship (Fig. 4*F*), suggesting that the concentration of aggregate_450nm_ causes proportional cell damage within this range of concentrations. Our data therefore imply that aggregate_450nm_ contains the most toxic species.

### AT630 Stains Pathological Aggregates Derived from PD and DLB.

**Fig. 5.**
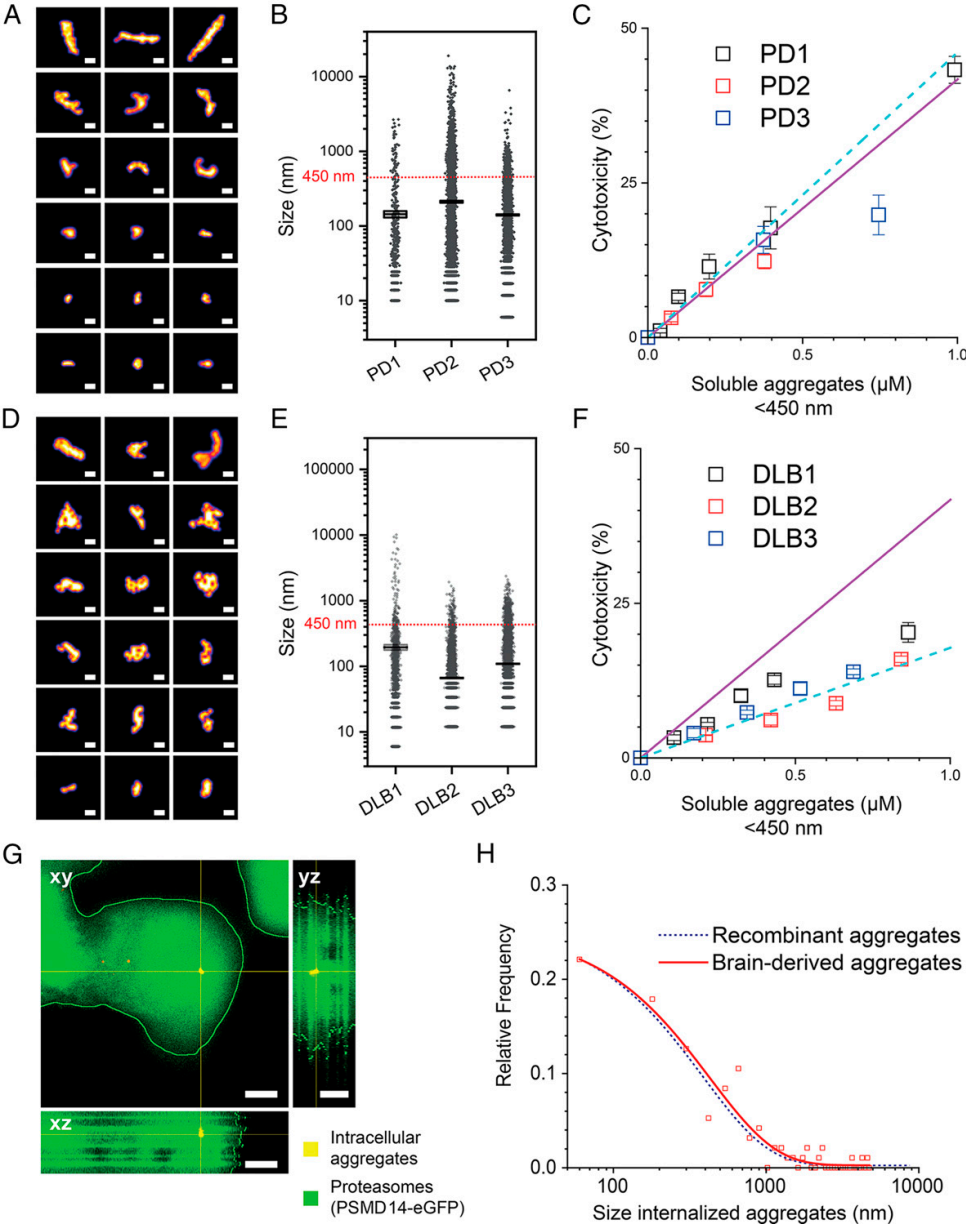
Aggregates derived from postmortem PD and DLB donors are quantitively detected by SMLM using AT630 staining. (*A*) Features of typical small aggregates detected in PD samples are shown. Scale bar represents 100 nm. (*B*) Quantification of superresolved aggregates by size in each donor sample is shown, with mean ± SEM sizes of 140 ± 10 nm, 211 ± 7 nm, and 106 ± 2 nm for PD1, PD2, and PD3, respectively (N_PD1_ = 599; N_PD2_ = 10,084; N_PD3_ = 8,663). The horizontal dashed line shows the 450 nm size threshold calculated in Fig. 3*B*. (*C*) LDH assay performed similar to HEK293A experiment in Fig. 4*F*, where cytotoxicity values are plotted against the calculated concentration of aggregate_450nm_. Increasing concentrations of PD samples were used to determine the linear range (see also *SI Appendix*, Fig. S11), which is plotted here, and are fitted to a straight line (cyan dashed line). The plotted values are in agreement with the toxicity-to-aggregate_450nm_ relationship determined in Fig. 4*F* (solid magenta line). The fraction of aggregate_450nm_ found in the samples for PD1, PD2, and PD3 were 11%, 12%, and 20%, respectively. (*D*) Features of typical aggregates detected in DLB samples are shown. Scale bar represents 100 nm. (*E*) Quantification of super-resolved aggregates by size in each donor sample is shown, with mean sizes ± SEM of 200 ± 20 nm, 66 ± 2 nm, and 109 ± 2 nm for DLB1, DLB2, and DLB3 samples, respectively (N_DLB1_ = 1,446; N_DLB2_ = 8,009; N_DLB3_ = 11,866). The horizontal dashed line shows the 450 nm size threshold calculated in Fig. 3*B*. (*F*) Cytotoxicity levels of three DLB samples plotted against the concentration of aggregate_450nm_. The solid magenta line represents the linear relationship in Fig. 4*F* determined from recombinant αS samples, and the cyan dashed line represents a linear relationship of the DLB samples. The fraction of aggregate_450nm_ in DLB1, DLB2, and DLB3 is 43%, 84%, and 69%, respectively. (*G*) Live HEK293A cells expressing PSMD14-eGFP (green) were also incubated with 1 μM PD1 patient-derived aggregates and stained with AT630 (red). Scale bars represent 5 µm. (*H*) Quantification of PD1 aggregates within intracellular domain (N_PD1,4h_ = 4,740 aggregates inside 115 cells). The blue dashed line is taken from the analogous experiment shown in *B,* where cells were incubated with αS aggregates for 4 h. Error bars represent SD of triplicate measurements (n = 3).

To test our cytotoxicity–aggregate_450nm_ correlation established in Fig. 4*F*, we performed brain soaks on postmortem cortical brain tissues from three PD donors by modifying an existing protocol (76) (*SI Appendix*, Fig. S10). Following our optimized protocol, AT630 efficiently stained the extracted aggregates and enabled imaging by SMLM. Detailed structural features of typical tissue-derived aggregate_450nm_ are shown in Fig. 5*A*. All three PD samples contained predominantly small aggregate species with mean sizes ± SEM of 140 ± 10 nm, 211 ± 7 nm, and 106 ± 2 nm for PD1, PD2, and PD3, respectively (Fig. 5*B* and Video S7). We used SMLM to calculate the fraction of aggregate_450nm_ in all three samples (11%, 12%, and 20%, respectively) by applying similar calculations as in Fig. 4, from which we then determined the estimated aggregate_450nm_ concentration. Cytotoxicity was next measured for increasing concentrations of each sample and plotted against the aggregate_450nm_ concentration (*SI Appendix*, Fig. S11). At higher sample concentrations of brain-derived aggregate_450nm_, the amount of aggregates larger than 450 nm also increases and may interfere with the potency of aggregates_450nm_, potentially perturbing the linear relationship with LDH cytotoxicity. However, within the linear sections of each cytotoxicity curve, we observed slopes that were largely in agreement with the cytotoxicity–aggregate_450nm_ relationship in Fig. 4*F* with gradients of 42 ± 6 μM^−1^ and 46 ± 4 μM^−1^ for recombinant αS and PD samples, respectively (Fig. 5*C* and *SI Appendix*, Fig. S11).

We subsequently investigated if aggregate_450nm_ species derived from postmortem brain tissues of three donors with DLB also obeyed the same correlation with size and cytotoxicity (Fig. 5 *D*–*F*). Examining the size distribution, the mean ± SEM aggregate sizes for DLB samples were 200 ± 20 nm, 66 ± 2 nm and 109 ± 2 nm, respectively, and smaller than those observed for PD. DLB samples also contained high fractions of aggregate_450nm_ within each mixture (43%, 84% and 69%, respectively). While the corresponding cytotoxicity values do not fall on the same linear slope shared by the recombinant αS and PD samples, they remain linearly correlated with aggregate_450nm_ concentration with a gradient of 17.8 ± 0.6 μM^−1^ (Fig. 5*F*).

We further compared the cytotoxicity of 1 μM PD and DLB samples after 4 and 24 h (*SI Appendix*, Fig. S12*A*) and found no significant difference between the two incubation periods, supporting the view that the aggregates that penetrate cells at 4 h are responsible for the observed toxicity. The cytotoxicity level did not depend on the effective concentration of fibrils larger than 450 ± 60 nm but on small aggregates (*SI Appendix*, Fig. S12*B*), suggesting that any toxic effects resulting from larger fibrils in the brain may involve more complex pathways *in vivo*. This agrees with our previous results (29) and also supports the conclusion that the size, rather than concentration of total aggregates, determines their innate ability to harm cells. To validate the toxicity of small aggregates, we incubated 1 μM PD patient–derived aggregates (PD1) with HEK293A cells expressing PSMD14-eGFP for 4 h. The distribution of PD1 aggregate sizes inside cells was similar to that of intracellular recombinant αS aggregates found in Fig. 3 (Fig. 5 *G* and *H*). This supports the observed similarity in toxicity gradient of recombinant and PD aggregates in Fig. 5*C*, again confirming that the small aggregate species exert toxicity by penetrating the plasma membrane.

Together, our cytotoxicity data demonstrate that the αS aggregates present in different synucleinopathies may possess distinct potency in causing cell damage. This could be important to explain why different pathologies arise from misfolding and aggregation of the same protein. It will be interesting to explore how aggregate size and other structural features influence spreading in different cell types to fully understand the activity of aggregates in each disease. Our findings validate the robustness of using the SMLM approach to determine cytotoxicity based on the fraction of aggregate_450nm_ within a heterogeneous aggregate sample. Our SMLM approach may further be applied to other biological samples and fluids, through which toxicity may be determined in a similar manner.

### AT630 Stains Aggregate Species in Mouse Brain Tissues.

**Fig. 6.**
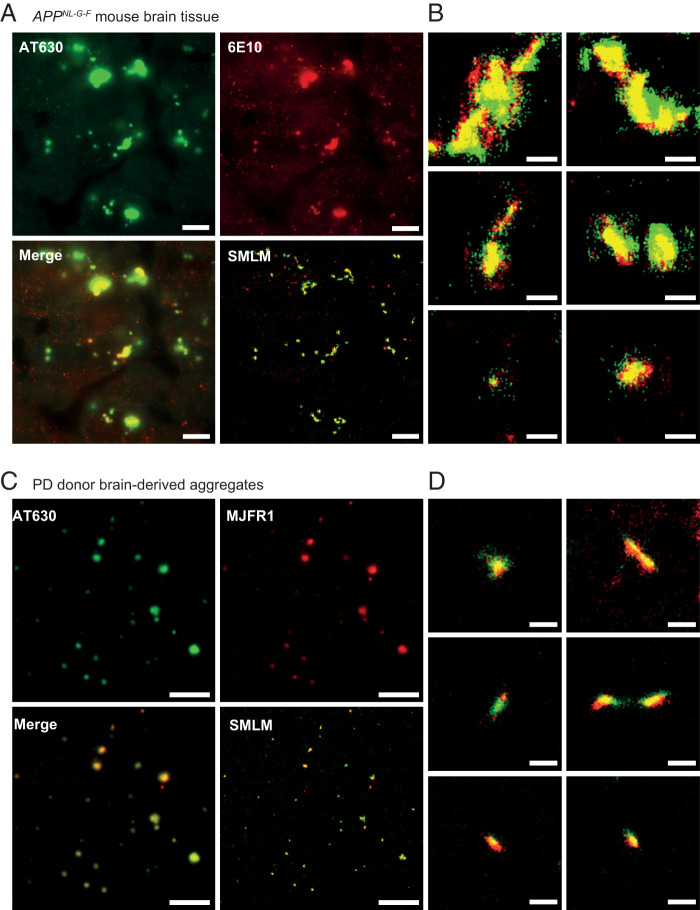
AT630 enables SMLM imaging of aggregates in *App^NL-G-F^* mouse brain tissues and human brain-derived aggregates. (*A*) Tissues are stained with AT630 (green, *Top Left*), anti-Aβ antibody 6E10 (red, *Top Right*); the merged image detected by diffraction-limited microscopy (*Bottom Left*) and the same merged image are shown super-resolved (*Bottom Right*) (see Video S7). Scale bars represent 5 μm. (*B*) Typical aggregates detected in *A* are shown. Scale bars represent 500 nm. (*C*) Aggregates isolated from the PD1 sample were imaged on a coverslip stained with both AT630 (green, *Top Left*) and anti-αS antibodies (red, *Top Right*), with the merged image (*Bottom Left*) super-resolved (*Bottom Right*). Scale bars represent 5 μm. (*D*) Typical aggregates from *C* are shown. Scale bars represent 500 nm.

We further validated the ability of AT630 to characterize pathological aggregates in situ and tested our SMLM approach on brain tissues from transgenic *App^NL-G-F^* mice. These mice are genetically engineered to express mutant APP proteins that are then processed into aggregation-prone Aβ42 peptides, leading to aggregate formation in the brain (77). To confirm the presence of pathological aggregates in the tissue, we performed immunostaining using 6E10, an Aβ-specific antibody, together with AT630 staining. Tissues were sectioned in 10 μm slices and imaged under HILO conditions, which produced background fluorescence in both channels using diffraction-limited imaging methods (Fig. 6 *A* and *B*). Regions with amyloid structures in the tissue were appreciable by both antibody and AT630 staining, albeit offering little information about aggregate features (in red and green, respectively, Fig. 6*A*). To examine tissue-bound aggregates in detail, we performed SMLM to reveal the distinct aggregate structures within the amyloid regions and demonstrated a very high degree of colocalization between AT630 and the 6E10 antibody (Fig. 6*B* and Video S7). We quantified the percentage of colocalization by dividing the total number of colocalized signals with the total AT630 signal (see *Materials and Methods*) and found 70 ± 20% agreement between AT630 and the antibody. Considering the increased noise level associated with tissues, the significant overlap underscores the specificity of AT630 in aggregate detection. In support of its specificity for aggregates, AT630 staining of aggregates derived from PD brain soak colocalized with anti-αS antibody (MJFR1) with 70 ± 5% (Fig. 6 *C* and *D*). Of note is the benefit of using azimuthal or spinning TIRF image averaging to improve signal-to-background in tissue imaging (*SI Appendix*, Fig. S13).

## Conclusions

For this study, we developed an SMLM approach using aggregate-activated fluorophores ProteoStat and AT630, which enabled characterization of aggregates by their size and detection of structural features of small aggregates down to 10 nm in size. Our data suggest that both ProteoStat and AT630 sensitively recognize features in smaller aggregate species in addition to fibrils. Combined with the observation that ThT does not seem to impede ProteoStat and AT630 staining, it is plausible that all three fluorophores recognize distinct features within aggregate structures.

Our approach enables quantitative detection of aggregates and especially aggregate_450nm_ as a distinct species and demonstrates their increased cytotoxicity associated with cell permeabilization. Using HEK293A cells, these invasive aggregates can be distinguished from fibrils, which are prevented from entering the cell by the plasma membrane. We further provide evidence that membrane-permeabilizing aggregates are targeted inside the cell by proteasomes, which concentrate locally into foci, probably as part of wider mechanisms to remove harmful agents (74). Given their size, it is possible that aggregate_450nm_ may be fragments of amyloid fibrils. This would be in line with our previous report of higher toxicity of fibrils fragmented by the proteasome (29) and consistent with recent studies describing self-replication of amyloid fibrils through fragmentation (78). Since AT630 is suitable for aggregate staining in live cells, employing this aggregate-proteasome assay in conjunction with quantitative SMLM of aggregates could be a combined approach to determine the level of aggregate_450nm_ and their toxicity within a biological sample. Such strategies would be beneficial for early-stage disease detection of proteopathies, for example, and the difference in linear relationships between cytotoxicity and aggregate_450nm_ concentration (Fig. 5 *C* and *F*) could enable diagnosis of specific diseases.

Importantly, our data on PD and DLB suggest that a population of small αS aggregates (aggregate_450nm_) from different synucleinopathies may have distinct toxic effects upon cells. This could perhaps be related to specific conformations assumed by the aggregates in each pathology and in line with the observed variations in structural conformations of αS fibrils reported for different synucleinopathies and for recombinant αS fibrils (79–81). Another possibility may be that the toxicity is proportional to the number of constituent misfolded protein monomers the individual aggregates contain. A previous study suggested no apparent correlation between the size of small aggregates and toxicity (17). The size of aggregates as observed by current imaging techniques do not necessarily correlate proportionally with the number of constituent monomers. Therefore, although our data suggest that stable fibrils are less toxic than small aggregates, it remains to be determined whether it is the aggregate_450nm_ size or their constituent monomer number that critically correlates with the observed toxicity. Nevertheless, the linear relationship between size and cytotoxicity in all samples indicate that size is a crucial factor determining membrane permeability for aggregates, while the conformations that aggregates adopt may also influence toxicity once inside the cell.

The quantitative imaging approach we developed here will facilitate further studies on understanding the cellular mechanisms underlying neurodegenerative disorders. For example, the methods outlined here offer a highly sensitive technique to study brain tissue, with the potential to identify cell types vulnerable to invasion by aggregates from the early stages of disease and beyond. Soluble aggregates have critical roles in propagating aggregate growth between cells (12); they are also faster at being internalized than fibrils, and are more likely to seed further aggregation events (82–84). Incomplete aggregate disassembly by proteasomes inside the cell can also increase the probability of aggregate elongation and further rationalizes the increased toxicity and spreading of aggregates that we and others have observed (29, 47, 85, 86). Using ProteoStat and AT630, which we now regard as the next generation of fluorophores, we uncovered aggregate species that are abundant at earlier stages of aggregation but were not detected by fluorophores traditionally used in imaging strategies. We have demonstrated that these aggregate-activated fluorophores may be used to quantify pathogenic aggregates, paving the path for future molecular studies relevant to neurodegeneration.

## Materials and Methods

### Preparation and Aggregation of Misfolded Proteins.

Purification of recombinant full-length tau P301S mutant (isoform 0N4R) and αS followed protocols others and we have described before (29, 87). Lyophilized synthetic Aβ42 (4014447; Bachem) was dissolved in phosphate-buffered saline (PBS) containing 0.5 M NaOH to make stock solution at 10 mM.

Proteins were aggregated according to standard protocols described elsewhere (17, 29).

Aggregation of 2 µM tau in PBS was induced with an equimolar final concentration of low-molecular-weight heparin (5 kDa; Thermo Fisher Scientific) at 37 °C for 24 h. Aβ42 aggregation was performed at 10 µM final concentration at 37 °C in reaction buffer (50 mM Tris and 150 mM NaCl, pH 7.4). For αS aggregation, the protein was diluted to 70 µM final concentration in reaction buffer containing 0.1% NaN_3_ and incubated at 37 °C, 200 rpm for 72 h. Formation of fibrils from the aggregation reaction was verified by ThT on a Cary Eclipse spectrophotometer.

### Extraction of Human Soluble Aggregates.

Soluble aggregates were extracted from the brain tissues of PD and DLB donors (*SI Appendix*, Table S1), following the protocol as described in Hong et al. (76). The specimens were obtained from the London Neurodegenerative Diseases Brain Bank, Brains for Dementia Research, and the Multiple Sclerosis and Parkinson’s Tissue Bank. Frozen temporal cortical tissues (∼0.5 g) were diced and incubated with artificial cerebrospinal fluid (aCSF) (124 mM NaCl, 2.8 mM KCl, 1.25 mM NaH_2_PO_4_, and 26 mM NaHCO_3_, pH 7.4) supplemented with protease inhibitors (A32953; Thermo Fisher Scientific) for 30 min at 4 °C. Samples were centrifuged for 10 min at 2,000 × *g*, and the supernatant was collected for subsequent ultracentrifugation at 200,000 × *g* for 110 min at 4 °C in a SW41 Ti rotor. The upper 80% of the resulting supernatant was dialyzed against a 100-fold excess of fresh aCSF for 72 h, with buffer changed every 24 h. Protein concentrations of the dialyzed samples were determined by Bradford assay, aliquoted, and stored at -80 °C.

### Mouse Tissue Staining.

Animal experiments were conducted according to the United Kingdom Animals (Scientific 643 Procedures) Act 1986. Brain hemispheres were flash frozen from adult (P240) *App^NL-G-F^* homozygous mice harboring the Swedish (KM670/671 N L), Beyreuther/Iberian (I716 F), and Arctic (E22G) mutations in the *APP* gene on a C57BL/J6 background. Animals were transcardially perfused with 20 mL ice-cold (4 °C) oxygenated dissection aCSF (108 mM choline-Cl, 3 mM KCl, 26 mM NaHCO3, 1.25 mM NaHPO4, 25 mM D-glucose, 3 mM Na pyruvate, 2 mM CaCl_2_, and 1 mM MgSO_4_ saturated with 95% O_2_/5% CO_2_). OCT embedding matrix was used to fix hemispheres, which were subsequently sectioned in 10 µm slices on a cryostat at -20 °C. Before staining, each section was repeatedly washed in PBS to remove the OCT, followed by incubation with PBS containing 4% paraformaldehyde, 0.3% Triton-X100. The same buffer was used for blocking with goat serum prior to staining with primary anti-Aβ antibody 6E10 overnight. Secondary Alexa488-tagged antibodies were used for staining the next day, followed by AT630 staining immediately before super-resolution imaging.

### Cytotoxicity Assay.

Toxicity of aggregates was determined with LDH assay (88). We previously described the procedure (29) for aggregate measurement. Aggregate samples or control buffers were added in triplicates to 200 μL cell media containing 30,000 HEK293A cells per well in 48-well plates. Lysis buffer was added 45 min before the end of the experiment to the maximum LDH activity set of control cells. After 4- or 24 h incubation at 37 °C, medium was removed and centrifuged at 200 × *g* for 5 min. Then, 50 µL media was incubated with 50 µL of the LDH assay buffer for 1 h, and the reactions were quenched with 50 μL 1 M acetic acid. Absorbance at 490 nm and 680 nm was measured for each reaction on a plate reader.

### Fluorophore Staining and Imaging.

ProteoStat (ENZ-51023; Enzo Lifesciences) or AT630 (Ebba Biotech) was diluted 1:50 in PBS for stock aliquots and stored at -20 °C. This stock was further diluted 1:50 (ProteoStat) or 1:1,000 (AT630) for final single-aggregate TIRF imaging. Single-aggregate TIRF imaging was performed as described before (29). Briefly, aggregates were diluted to 700 nM (αS), 400 nM (tau), or 300 nM (Aβ) imaging concentrations with indicated fluorophores and incubated on a coverslip for 15 min. Samples were imaged on a home-built TIRF microscope. For super-resolution microscopy, samples were incubated in freshly prepared imaging buffer [PBS with 1 mg/mL glucose oxidase, 0.02 mg/mL catalase, 10% (wt/wt) glucose, and 100 mM methylamine] immediately prior to imaging.

Generation of the HEK293A cells expressing eGFP-tagged *PSMD14* subunit is described in detail by Zhang et al. (64). Live-cell imaging of these cells incubated with 1 μM aggregates was conducted in FluoroBrite Dulbecco’s modified Eagle’s medium (Thermo Fisher Scientific) supplemented with 10% fetal bovine serum.

### Imaging with TIRF Microscope.

Samples were imaged using an ECLIPSE Ti2-E inverted microscope (Nikon). Lasers were housed in a C-FLEX laser combiner (HÜBNER Photonics), containing 405 nm (06 series, HÜBNER Photonics), 488 nm (06 series, HÜBNER Photonics), 561 nm (04 series HÜBNER Photonics), and 638 nm (06 series, HÜBNER Photonics) lasers. These were all aligned inside the combiner and were coupled to the E-TIRF arm (Nikon). A filter cube containing a dichroic mirror, in addition to bandpass and longpass filters for all four laser lines (C-NSTORM QUAD 405/488/561/647 FILT; Nikon) was installed inside a motorized filter turret below a CFI Apochromat TIRF 100XC NA 1.49 Oil objective (Nikon). Samples were placed on a motorized stage, and a Perfect Focusing System (Nikon) was used to minimize drift in the z direction. Images were recorded by an sCMOS camera (Prime95B, Photometrics) with a pixel size of 11 × 11 μm, at 20 Hz. Super-resolution (SMLM) images were reconstructed from 2,000 frames, and diffraction-limited images of aggregates on coverslips were averaged from 100 frames. In order to record axial information of single molecules, we placed a cylindrical lens (f = 1,000.0 mm, Plano-Convex; Thorlabs) in the Optosplit II and followed the astigmatism method of 3D SMLM (89).

### SMLM Image Reconstruction.

SMLM images in 2D were reconstructed using Matlab scripts described in detail by Yin et al. (90). Briefly, each frame was initially filtered using a box filter and set with a box four times the width of 2D Gaussian PSF, and each pixel intensity was weighted by the inverse of its variation. Local maxima were then recorded and fitted to a 2D-Gaussian single-PSF using the Maximum Likelihood Estimation algorithm performed in GPU (Nvidia GTX 1060, CUDA 8.0). Alignment of two colors was achieved by recording a map of chromatic aberrations using Tetraspeck beads (0.1 µm, fluorescent blue/green/orange/dark red; Life Technologies) that fluoresce across a broad range of wavelengths and readily adhere to a plasma-cleaned coverslip. The centers of these beads were recorded upon excitation of the 488 nm, 561 nm, and 638 nm lasers, and a second-order polynomial function was used to correct for the chromatic aberration across the three colors. For the calculation of localization precision, each burst of fluorescence from individual fluorophores was aligned relative to each other, and an average PSF was calculated. Calibration of the sCMOS camera enabled the offset for each pixel to be calculated and allowed the number of photons to be calculated for each burst (90). This was used to define the localization precision using $\Delta \mathrm{loc}=\mathrm{FWHM}/\sqrt{\mathrm{photons}}$.

Recorded 3D images and videos were reconstructed using ThunderSTORM (91). We again used the Tetraspeck beads deposited on a plasma-cleaned coverslip and imaged the beads while scanning along the z axis, with the cylindrical lens installed in the Optoslipt II. These images were used to produce reference curves that we used to calculate the axial position of the fluorophores bound to aggregates during our 3D SMLM experiments. Reconstruction of 3D models from SMLM were made by fitting the recorded images with the elliptical Gaussian PSF (astigmatism) model using the weighted least-squares method.

Following the methodology described by Huang et al. (89), we deposited αS monomers that were singly labeled with Alexa647 on a plasma-cleaned coverslip. We plotted the relative 2D and 3D positions measured within 100 nm from the average focal plane for each fluorophore across individual blinking cycles. This gave clusters of localizations that could be fitted to Gaussian curves to quantify our localization accuracy (*SI Appendix*, Fig. S4). SMLM in 2D gave Gaussians with an FWHM of 27 nm and 28 nm for both x and y directions. Adding the cylindrical lens resulted in Gaussians of 33 nm, 42 nm, and 84 nm for x, y, and z directions, respectively.

### Quantitative Analysis of Imaging Data.

Images produced from SMLM and diffraction-limited TIRF microscopy experiments were analyzed using custom written Matlab codes to quantify the lengths (sizes) and total intensities of each aggregate, previously described in detail by Cliffe et al. (29). Briefly, 100 frames were averaged, and the averaged images were top-hat and bpass filtered to reduce noise from the camera and subtract background. The resultant images were then blurred using a Gaussian filter before the outline of the individual aggregates was calculated. The boundaries were thinned to determine the size of each particle, and a signal-to-background correction for each pixel was used to measure the total intensities.

To identify aggregates that were internalized by cells, the whole cell was imaged using stacks of images 100 nm apart in the z direction. These cells expressed GFP-tagged proteasomes, and the cell boundaries from each stack were determined using a rolling ball filter on the images from the GFP emission (64). Aggregates that were found outside these boundaries were then masked from the stack of images and excluded from further analysis. The pixels were then summed in the z-projection across the stack of images, and these resulting 2D images were put through the size analysis described above.

Image analysis for colocalization experiments was performed using the Colocalization Threshold plug-in in ImageJ software. This analysis gave the thresholded Manders’ coefficients for each channel. The Colocalization Threshold plug-in uses the Costes method (92) to automatically determine the thresholds for the two channels.

### Statistical Calculations.

All uncertainties are presented to one significant figure only, as the second significant figure for each uncertainty would be within the error reported by the first. Similarly, the accuracy of all quantities is reported within its corresponding error; therefore, the last significant figure of each measurement is the same figure as the first significant figure in the corresponding uncertainty.

N numbers for the number of aggregates and cells imaged from each experiment are found in the corresponding figure legends. A Kolmogorov-Smirnov test was used in Fig. 1*D* to demonstrate that AT630 and ProteoStat produced a distribution of images that was statistically different to those observed using ThT (*P* < 0.0005). Scatter plots in Figs. 3–6 are reported with the mean values and SEM of the aggregate sizes for each aggregate sample. The crossover at 450 ± 60 nm for the 3 h and 24 h samples shown in Fig. 3*B* was calculated by fitting each distribution to a single exponential decay using Eq. 1, where x is the aggregate size, A is the amplitude, and $1/t_{1}$ represents the decay rate.

[1] $$y=Ae^{\left[-x/t_{1}\right]}$$

The error reported at the crossover is the square root of the sum of the SEM for *t*_1_ of the two fitted curves. Similarly, the 21 ± 3 nm shift in Fig. 3*D* was calculated by subtracting the median value of each curve, and the error is the square root of the sum of the SEM of *t*_1_ of the two fitted curves. Error bars in the inset in Fig. 3*D* represent the SD of the mean number of aggregates in an individual cell. Statistical significance between size groups at different time points was calculated using a two-sample *t* test with n.s. (no significance) *P* > 0.05, *P < 0.05, and **P < 0.005. Cytotoxicity values in Figs. 5 and 6 and *SI Appendix*, Figs. S10 and S11 are reported as mean and SD of triplicate experiments.

### Calculation of Photophysical Properties.

The quantum yield (Φ) and extinction coefficient (ε_A_) of Alexa488, Alexa568, Alexa647, and ThT have been previously validated by other research groups and the dyes’ manufacturers. The precise properties of ProteoStat and AT630 are protected due to proprietary reasons. The manual from Enzo Life Sciences states that ProteoStat is ∼3 μM at the recommended final concentration, and this was used to calculate an extinction coefficient from the UV-vis (ultraviolet-visible) absorption spectrum. Ebba Biotech, who produced the Amytracker dyes, was able to inform us that the average molecular weight across the range of Amytracker dyes was 660 ±30 g/mol. The user manual from Ebba Biotech also states that the concentration of AT630 is 1 mg/mL, and this was then used to calculate the molar concentration of AT630 and enabled us to calculate the extinction coefficient from the UV-vis absorption spectrum. The excitation and emission profile of Alexa568 is similar to that of both ProteoStat and AT630, so we used an anti-rabbit secondary antibody labeled with Alexa568 (Invitrogen; A11011) as our reference to calculate the relative quantum yields of ProteoStat and AT630. We recorded the emission spectra from a range of concentrations of the fluorophore absorbances ranging from 0.1 to 0.9 AU (arbitrary units), in the presence of an excess of αS fibrils (10 μM). The emission profiles were integrated (I) and plotted against the absorbance (A) and were fitted to a straight line. The gradients of each linear plot were compared to the Alexa568, using Eq. 2 described by Wong et al. (93) using the quantum yield of Alexa568 (Φ*_R_* = 0.69) as a reference. The brightness of each dye was calculated simply using Brightness = Φ × ε_A_.

[2] $$\Phi_{S}=\Phi_{R}\left(\frac{I_{S}}{I_{R}}\right)\left(\frac{1-10^{-A_{R}}}{1-10^{-A_{S}}}\right){\left(\frac{n_{S}}{n_{R}}\right)}^{2}$$

where n = refractive index of the solution.

## Supplementary Material

Supplementary File

Supplementary File

Supplementary File

Supplementary File

Supplementary File

Supplementary File

Supplementary File

Supplementary File

## Data Availability

All study data are included in the article and/or supporting information.

## Change History

October 14, 2022: The legends and video files for Video S5 and Video S6 were mismatched; the video files for Video S5 and Video S6 have been swapped to address this error. The corresponding author’s name has been updated in the online version.

## References

[1] C. Soto, S. Pritzkow, Protein misfolding, aggregation, and conformational strains in neurodegenerative diseases. Nat. Neurosci. 21, 1332–1340 (2018). 30250260 10.1038/s41593-018-0235-9 PMC6432913

[2] C. A. Ross, M. A. Poirier, Protein aggregation and neurodegenerative disease. Nat. Med. 10 (suppl.), S10–S17 (2004). 15272267 10.1038/nm1066

[3] F. Chiti, C. M. Dobson, Protein misfolding, functional amyloid, and human disease. Annu. Rev. Biochem. 75, 333–366 (2006). 16756495 10.1146/annurev.biochem.75.101304.123901

[4] M. Goedert, M. Masuda-Suzukake, B. Falcon, Like prions: The propagation of aggregated tau and α-synuclein in neurodegeneration. Brain 140, 266–278 (2017). 27658420 10.1093/brain/aww230

[5] M. Goedert, D. S. Eisenberg, R. A. Crowther, Propagation of tau aggregates and neurodegeneration. Annu. Rev. Neurosci. 40, 189–210 (2017). 28772101 10.1146/annurev-neuro-072116-031153

[6] M. G. Iadanza, M. P. Jackson, E. W. Hewitt, N. A. Ranson, S. E. Radford, A new era for understanding amyloid structures and disease. Nat. Rev. Mol. Cell Biol. 19, 755–773 (2018). 30237470 10.1038/s41580-018-0060-8 PMC7617691

[7] A. L. Mahul-Mellier , The process of Lewy body formation, rather than simply α-synuclein fibrillization, is one of the major drivers of neurodegeneration. Proc. Natl. Acad. Sci. U.S.A. 117, 4971–4982 (2020). 32075919 10.1073/pnas.1913904117 PMC7060668

[8] L. I. Binder, A. L. Guillozet-Bongaarts, F. Garcia-Sierra, R. W. Berry, Tau, tangles, and Alzheimer’s disease. Biochim. Biophys. Acta 1739, 216–223 (2005). 15615640 10.1016/j.bbadis.2004.08.014

[9] A. Gustot , Amyloid fibrils are the molecular trigger of inflammation in Parkinson’s disease. Biochem. J. 471, 323–333 (2015). 26272943 10.1042/BJ20150617

[10] L. V. Kalia, S. K. Kalia, P. J. McLean, A. M. Lozano, A. E. Lang, α-Synuclein oligomers and clinical implications for Parkinson disease. Ann. Neurol. 73, 155–169 (2013). 23225525 10.1002/ana.23746 PMC3608838

[11] D. M. Walsh, D. J. Selkoe, Oligomers on the brain: The emerging role of soluble protein aggregates in neurodegeneration. Protein Pept. Lett. 11, 213–228 (2004). 15182223 10.2174/0929866043407174

[12] M. L. Choi, S. Gandhi, Crucial role of protein oligomerization in the pathogenesis of Alzheimer’s and Parkinson’s diseases. FEBS J. 285, 3631–3644 (2018). 29924502 10.1111/febs.14587

[13] M. Iljina , Quantifying co-oligomer formation by α-synuclein. ACS Nano 12, 10855–10866 (2018). 30371053 10.1021/acsnano.8b03575 PMC6262461

[14] T. K. Karamanos , Structural mapping of oligomeric intermediates in an amyloid assembly pathway. eLife 8, e46574 (2019). 31552823 10.7554/eLife.46574 PMC6783270

[15] S. L. Shammas , A mechanistic model of tau amyloid aggregation based on direct observation of oligomers. Nat. Commun. 6, 7025 (2015). 25926130 10.1038/ncomms8025 PMC4421837

[16] D. Pinotsi , Nanoscopic insights into seeding mechanisms and toxicity of α-synuclein species in neurons. Proc. Natl. Acad. Sci. U.S.A. 113, 3815–3819 (2016). 26993805 10.1073/pnas.1516546113 PMC4833232

[17] S. De , Different soluble aggregates of Aβ42 can give rise to cellular toxicity through different mechanisms. Nat. Commun. 10, 1541 (2019). 30948723 10.1038/s41467-019-09477-3 PMC6449370

[18] A. Drews , Inhibiting the Ca^2+^ influx induced by human CSF. Cell Rep. 21, 3310–3316 (2017). 29241555 10.1016/j.celrep.2017.11.057 PMC5745229

[19] D. I. Sideris , Soluble amyloid beta-containing aggregates are present throughout the brain at early stages of Alzheimer's disease. Brain Commun. 3, fcab147 (2021). 34396107 10.1093/braincomms/fcab147 PMC8361392

[20] A. N. Nilson , Tau oligomers associate with inflammation in the brain and retina of tauopathy mice and in neurodegenerative diseases. J. Alzheimers Dis. 55, 1083–1099 (2017). 27716675 10.3233/JAD-160912 PMC5147514

[21] C. Kim , Neuron-released oligomeric α-synuclein is an endogenous agonist of TLR2 for paracrine activation of microglia. Nat. Commun. 4, 1562 (2013). 23463005 10.1038/ncomms2534 PMC4089961

[22] G. Meisl, T. P. Knowles, D. Klenerman, The molecular processes underpinning prion-like spreading and seed amplification in protein aggregation. Curr. Opin. Neurobiol. 61, 58–64 (2020). 32092527 10.1016/j.conb.2020.01.010

[23] S. W. Chen , Structural characterization of toxic oligomers that are kinetically trapped during α-synuclein fibril formation. Proc. Natl. Acad. Sci. 112, E1994–E2003 (2015). 25855634 10.1073/pnas.1421204112 PMC4413268

[24] N. Cremades , Direct observation of the interconversion of normal and toxic forms of α-synuclein. Cell 149, 1048–1059 (2012). 22632969 10.1016/j.cell.2012.03.037 PMC3383996

[25] P. Alam, L. Bousset, R. Melki, D. E. Otzen, α-Synuclein oligomers and fibrils: A spectrum of species, a spectrum of toxicities. J. Neurochem. 150, 522–534 (2019). 31254394 10.1111/jnc.14808

[26] J. Habchi , Cholesterol catalyses Aβ42 aggregation through a heterogeneous nucleation pathway in the presence of lipid membranes. Nat. Chem. 10, 673–683(2018). 29736006 10.1038/s41557-018-0031-x

[27] T. V. Kamath , Kinetics of tau aggregation reveals patient-specific tau characteristics among Alzheimer's cases. Brain Commun. 3, fcab096 (2021). 34222869 10.1093/braincomms/fcab096 PMC8244646

[28] H. Wesseling , Tau PTM profiles identify patient heterogeneity and stages of Alzheimer’s disease. Cell 183, 1699–1713.e13 (2020). 33188775 10.1016/j.cell.2020.10.029 PMC8168922

[29] R. Cliffe , Filamentous aggregates are fragmented by the proteasome holoenzyme. Cell Rep. 26, 2140–2149.e3 (2019). 30784595 10.1016/j.celrep.2019.01.096 PMC6381791

[30] Y. Ye, D. Klenerman, D. Finley, N-terminal ubiquitination of amyloidogenic proteins triggers removal of their oligomers by the proteasome holoenzyme. J. Mol. Biol. 432, 585–596 (2020). 31518613 10.1016/j.jmb.2019.08.021 PMC6990400

[31] H. B. Rajamohamedsait, E. M. Sigurdsson, Histological staining of amyloid and pre-amyloid peptides and proteins in mouse tissue. Methods Mol. Biol. 849, 411–424 (2012). 22528106 10.1007/978-1-61779-551-0_28 PMC3859432

[32] C. Xue, T. Y. Lin, D. Chang, Z. Guo, Thioflavin T as an amyloid dye: Fibril quantification, optimal concentration and effect on aggregation. R. Soc. Open Sci. 4, 160696 (2017). 28280572 10.1098/rsos.160696 PMC5319338

[33] M. Biancalana, S. Koide, Molecular mechanism of Thioflavin-T binding to amyloid fibrils. Biochim. Biophys. Acta 1804, 1405–1412 (2010). 20399286 10.1016/j.bbapap.2010.04.001 PMC2880406

[34] J. Ries , Superresolution imaging of amyloid fibrils with binding-activated probes. ACS Chem. Neurosci. 4, 1057–1061 (2013). 23594172 10.1021/cn400091m PMC3715833

[35] H. A. Shaban, C. A. Valades-Cruz, J. Savatier, S. Brasselet, Polarized super-resolution structural imaging inside amyloid fibrils using Thioflavine T. Sci. Rep. 7, 12482 (2017). 28970520 10.1038/s41598-017-12864-9 PMC5624930

[36] J. Torra, P. Bondia, S. Gutierrez-Erlandsson, B. Sot, C. Flors, Long-term STED imaging of amyloid fibers with exchangeable Thioflavin T. Nanoscale 12, 15050–15053 (2020). 32666991 10.1039/d0nr02961k

[37] L.-M. Needham , ThX - A next-generation probe for the early detection of amyloid aggregates. Chem. Sci. (Camb.) 11, 4578–4583 (2020). 10.1039/c9sc04730a PMC8159457 34122915

[38] J.-E. Lee , Mapping surface hydrophobicity of α-synuclein oligomers at the nanoscale. Nano Lett. 18, 7494–7501 (2018). 30380895 10.1021/acs.nanolett.8b02916 PMC6295917

[39] I. Maezawa , Congo red and thioflavin-T analogs detect Aβ oligomers. J. Neurochem. 104, 457–468 (2008). 17953662 10.1111/j.1471-4159.2007.04972.x

[40] F. Kundel , Shedding light on aberrant interactions - A review of modern tools for studying protein aggregates. FEBS J. 285, 3604–3630 (2018). 29453901 10.1111/febs.14409

[41] F. S. Ruggeri, J. Habchi, A. Cerreta, G. Dietler, AFM-based single molecule techniques: Unraveling the amyloid pathogenic species. Curr. Pharm. Des. 22, 3950–3970 (2016). 27189600 10.2174/1381612822666160518141911 PMC5080865

[42] M. H. Horrocks , Fast flow microfluidics and single-molecule fluorescence for the rapid characterization of α-synuclein oligomers. Anal. Chem. 87, 8818–8826 (2015). 26258431 10.1021/acs.analchem.5b01811

[43] M. Iljina , Arachidonic acid mediates the formation of abundant alpha-helical multimers of alpha-synuclein. Sci. Rep. 6, 33928 (2016). 27671749 10.1038/srep33928 PMC5037366

[44] G. T. Dempsey, J. C. Vaughan, K. H. Chen, M. Bates, X. Zhuang, Evaluation of fluorophores for optimal performance in localization-based super-resolution imaging. Nat. Methods 8, 1027–1036 (2011). 22056676 10.1038/nmeth.1768 PMC3272503

[45] F. Kundel , Hsp70 inhibits the nucleation and elongation of tau and sequesters tau aggregates with high affinity. ACS Chem. Biol. 13, 636–646 (2018). 29300447 10.1021/acschembio.7b01039 PMC6374916

[46] D. R. Whiten , Nanoscopic characterisation of individual endogenous protein aggregates in human neuronal cells. ChemBioChem 19, 2033–2038 (2018). 30051958 10.1002/cbic.201800209 PMC6220870

[47] E. Lobanova, , Imaging protein aggregates in the serum and cerebrospinal fluid in Parkinson’s disease. Brain, 145, 632–643 (2022). 34410317 10.1093/brain/awab306 PMC9014748

[48] F. S. Ruggeri, T. Šneideris, M. Vendruscolo, T. P. J. Knowles, Atomic force microscopy for single molecule characterisation of protein aggregation. Arch. Biochem. Biophys. 664, 134–148 (2019). 30742801 10.1016/j.abb.2019.02.001 PMC6420408

[49] D. Shen , Novel cell- and tissue-based assays for detecting misfolded and aggregated protein accumulation within aggresomes and inclusion bodies. Cell Biochem. Biophys. 60, 173–185 (2011). 21132543 10.1007/s12013-010-9138-4 PMC3112480

[50] D. Sehlin, X. T. Fang, S. R. Meier, M. Jansson, S. Syvänen, Pharmacokinetics, biodistribution and brain retention of a bispecific antibody-based PET radioligand for imaging of amyloid-β. Sci. Rep. 7, 17254 (2017). 29222502 10.1038/s41598-017-17358-2 PMC5722892

[51] F. J. B. Bäuerlein , In situ architecture and cellular interactions of polyQ inclusions. Cell 171, 179–187.e110 (2017). 28890085 10.1016/j.cell.2017.08.009

[52] F. Frottin , The nucleolus functions as a phase-separated protein quality control compartment. Science 365, 342–347 (2019). 31296649 10.1126/science.aaw9157

[53] M. Calvo-Rodriguez , In vivo detection of tau fibrils and amyloid β aggregates with luminescent conjugated oligothiophenes and multiphoton microscopy. Acta Neuropathol. Commun. 7, 171 (2019). 31703739 10.1186/s40478-019-0832-1 PMC6839235

[54] N. P. Cook, K. Kilpatrick, L. Segatori, A. A. Martí, Detection of α-synuclein amyloidogenic aggregates in vitro and in cells using light-switching dipyridophenazine ruthenium(II) complexes. J. Am. Chem. Soc. 134, 20776–20782 (2012). 23237404 10.1021/ja3100287

[55] D. Laor , Fibril formation and therapeutic targeting of amyloid-like structures in a yeast model of adenine accumulation. Nat. Commun. 10, 62 (2019). 30622276 10.1038/s41467-018-07966-5 PMC6325136

[56] J. A. Varela , Optical structural analysis of individual α-synuclein oligomers. Angew. Chem. Int. Ed. Engl. 57, 4886–4890 (2018). 29342318 10.1002/anie.201710779 PMC5988047

[57] K. Spehar , Super-resolution imaging of amyloid structures over extended times by using transient binding of single Thioflavin T molecules. ChemBioChem 19, 1944–1948 (2018). 29953718 10.1002/cbic.201800352 PMC6428420

[58] S. Wegmann , Tau protein liquid-liquid phase separation can initiate tau aggregation. EMBO J. 37, e98049 (2018). 29472250 10.15252/embj.201798049 PMC5881631

[59] B. Huang, S. A. Jones, B. Brandenburg, X. Zhuang, Whole-cell 3D STORM reveals interactions between cellular structures with nanometer-scale resolution. Nat. Methods 5, 1047–1052 (2008). 19029906 10.1038/nmeth.1274 PMC2596623

[60] M. J. Rust, M. Bates, X. Zhuang, Sub-diffraction-limit imaging by stochastic optical reconstruction microscopy (STORM). Nat. Methods 3, 793–795 (2006). 16896339 10.1038/nmeth929 PMC2700296

[61] E. Betzig , Imaging intracellular fluorescent proteins at nanometer resolution. Science 313, 1642–1645 (2006). 16902090 10.1126/science.1127344

[62] S. T. Hess, T. P. Girirajan, M. D. Mason, Ultra-high resolution imaging by fluorescence photoactivation localization microscopy. Biophys. J. 91, 4258–4272 (2006). 16980368 10.1529/biophysj.106.091116 PMC1635685

[63] R. J. Karpowicz Jr., J. Q. Trojanowski, V. M. Lee, Transmission of α-synuclein seeds in neurodegenerative disease: Recent developments. Lab. Invest. 99, 971–981 (2019). 30760864 10.1038/s41374-019-0195-z PMC6609465

[64] Y. Zhang , Membrane potential regulates the dynamic localisation of mammalian proteasomes. *bioRxiv* [Preprint] (2018). 10.1101/487702. Accessed 15 September 2022.

[65] L. Lesire , High-throughput image-based aggresome quantification. SLAS Discov. 25, 783–791 (2020). 32449635 10.1177/2472555220919708

[66] M. Tokunaga, N. Imamoto, K. Sakata-Sogawa, Highly inclined thin illumination enables clear single-molecule imaging in cells. Nat. Methods 5, 159–161 (2008). 18176568 10.1038/nmeth1171

[67] S. Yasuda , Stress- and ubiquitylation-dependent phase separation of the proteasome. Nature 578, 296–300 (2020). 32025036 10.1038/s41586-020-1982-9

[68] W. Qiang, K. Kelley, R. Tycko, Polymorph-specific kinetics and thermodynamics of β-amyloid fibril growth. J. Am. Chem. Soc. 135, 6860–6871 (2013). 23627695 10.1021/ja311963f PMC3686096

[69] E. Lindersson , Proteasomal inhibition by α-synuclein filaments and oligomers. J. Biol. Chem. 279, 12924–12934 (2004). 14711827 10.1074/jbc.M306390200

[70] H. J. Lee , Direct transfer of α-synuclein from neuron to astroglia causes inflammatory responses in synucleinopathies. J. Biol. Chem. 285, 9262–9272 (2010). 20071342 10.1074/jbc.M109.081125 PMC2838344

[71] H. J. Lee, S. J. Lee, Characterization of cytoplasmic α-synuclein aggregates. Fibril formation is tightly linked to the inclusion-forming process in cells. J. Biol. Chem. 277, 48976–48983 (2002). 12351642 10.1074/jbc.M208192200

[72] Q. Guo , In situ structure of neuronal C9orf72 poly-GA aggregates reveals proteasome recruitment. Cell 172, 696–705.e12 (2018). 29398115 10.1016/j.cell.2017.12.030 PMC6035389

[73] R. Hao , Proteasomes activate aggresome disassembly and clearance by producing unanchored ubiquitin chains. Mol. Cell 51, 819–828 (2013). 24035499 10.1016/j.molcel.2013.08.016 PMC3791850

[74] E. Mee Hayes, L. Sirvio, Y. Ye, A potential mechanism for targeting aggregates with proteasomes and disaggregases in liquid droplets. Front. Aging Neurosci. 14, 854380 (2022). 35517053 10.3389/fnagi.2022.854380 PMC9062979

[75] A. Marreiro , Comparison of size distribution and (Pro249-Ser258) epitope exposure in in vitro and in vivo derived Tau fibrils. BMC Mol. Cell Biol. 21, 81 (2020). 33183222 10.1186/s12860-020-00320-y PMC7661158

[76] W. Hong , Diffusible, highly bioactive oligomers represent a critical minority of soluble Aβ in Alzheimer’s disease brain. Acta Neuropathol. 136, 19–40 (2018). 29687257 10.1007/s00401-018-1846-7 PMC6647843

[77] T. Saito , Single *App* knock-in mouse models of Alzheimer’s disease. Nat. Neurosci. 17, 661–663 (2014). 24728269 10.1038/nn.3697

[78] G. Meisl , Uncovering the universality of self-replication in protein aggregation and its link to disease. Sci. Adv. 8, eabn6831 (2022). 35960802 10.1126/sciadv.abn6831 PMC9374340

[79] M. Schweighauser , Structures of α-synuclein filaments from multiple system atrophy. Nature 585, 464–469 (2020). 32461689 10.1038/s41586-020-2317-6 PMC7116528

[80] R. Guerrero-Ferreira , Cryo-EM structure of alpha-synuclein fibrils. eLife 7, e36402 (2018). 29969391 10.7554/eLife.36402 PMC6092118

[81] Y. Sun , Cryo-EM structure of full-length α-synuclein amyloid fibril with Parkinson’s disease familial A53T mutation. Cell Res. 30, 360–362 (2020). 32203130 10.1038/s41422-020-0299-4 PMC7118165

[82] T. Yang, S. Li, H. Xu, D. M. Walsh, D. J. Selkoe, Large soluble oligomers of amyloid β-protein from Alzheimer brain are far less neuroactive than the smaller oligomers to which they dissociate. J. Neurosci. 37, 152–163 (2017). 28053038 10.1523/JNEUROSCI.1698-16.2016 PMC5214627

[83] G. Fusco , Structural basis of membrane disruption and cellular toxicity by α-synuclein oligomers. Science 358, 1440–1443 (2017). 29242346 10.1126/science.aan6160

[84] M. Usenovic , Internalized tau oligomers cause neurodegeneration by inducing accumulation of pathogenic tau in human neurons derived from induced pluripotent stem cells. J. Neurosci. 35, 14234–14250 (2015). 26490863 10.1523/JNEUROSCI.1523-15.2015 PMC6605424

[85] J. H. Brelstaff , Microglia become hypofunctional and release metalloproteases and tau seeds when phagocytosing live neurons with P301S tau aggregates. Sci. Adv. 7, eabg4980 (2021). 34669475 10.1126/sciadv.abg4980 PMC8528424

[86] P. d’Errico , Microglia contribute to the propagation of Aβ into unaffected brain tissue. Nat. Neurosci. 25, 20–25 (2022). 34811521 10.1038/s41593-021-00951-0 PMC8737330

[87] C. Huang, G. Ren, H. Zhou, C. C. Wang, A new method for purification of recombinant human α-synuclein in *Escherichia coli*. Protein Expr. Purif. 42, 173–177 (2005). 15939304 10.1016/j.pep.2005.02.014

[88] S. Kaja , An optimized lactate dehydrogenase release assay for screening of drug candidates in neuroscience. J. Pharmacol. Toxicol. Methods 73, 1–6 (2015). 25681780 10.1016/j.vascn.2015.02.001 PMC4458191

[89] B. Huang, W. Wang, M. Bates, X. Zhuang, Three-dimensional super-resolution imaging by stochastic optical reconstruction microscopy. Science 319, 810–813 (2008). 18174397 10.1126/science.1153529 PMC2633023

[90] Y. Yin, W. T. C. Lee, E. Rothenberg, Ultrafast data mining of molecular assemblies in multiplexed high-density super-resolution images. Nat. Commun. 10, 119 (2019). 30631072 10.1038/s41467-018-08048-2 PMC6328550

[91] M. Ovesný, P. Křížek, J. Borkovec, Z. Svindrych, G. M. Hagen, ThunderSTORM: A comprehensive ImageJ plug-in for PALM and STORM data analysis and super-resolution imaging. Bioinformatics 30, 2389–2390 (2014). 24771516 10.1093/bioinformatics/btu202 PMC4207427

[92] S. V. Costes , Automatic and quantitative measurement of protein-protein colocalization in live cells. Biophys. J. 86, 3993–4003 (2004). 15189895 10.1529/biophysj.103.038422 PMC1304300

[93] K. L. Wong, J. C. G. Bunzli, P. A. Tanner, Quantum yield and brightness. J. Lumin. 224, 117256 (2020).

