# Supplementary material for: Quantitative super-resolution imaging of pathological aggregates reveals distinct toxicity profiles in different synucleinopathies
Source: Proc Natl Acad Sci U S A. 2022 Oct 7;119(41):e2205591119. doi: 10.1073/pnas.2205591119 (PMC9573094; doi:10.1073/pnas.2205591119)
Supplement: Supplementary File [file pnas.2205591119.sapp.pdf]

Supplementary Information

Quantitative super-resolution imaging of pathological aggregates reveals distinct toxicity profiles in different synucleinopathies

Michael J. Morten,[1,2] Liina Sirvio[1,2], Huzefa Rupawala[1,2], Emma Mee Hayes[1,2], Aitor Franco[3], Carola Radulescu[1,2], Liming Ying[4], Samuel J. Barnes[1,2], Arturo Muga[3], Yu Ye.[1,2,*]

[1]Department of Brain Sciences, Imperial College London, W12 0NN, UK
[2]UK Dementia Research Institute at Imperial College London, W12 0BZ, UK
[3]Instituto Biofisika (UPV/EHU, CSIC), University of the Basque Country, 48940 Leioa, Spain
[4]National Heart and Lung Institute, Imperial College London, W12 0BZ, UK

Email: yu.ye1@imperial.ac.uk

Figure S1. Photophysical properties of ProteoStat and AT630. **(A)** UV-visible absorption and emission spectra of ThT, ProteoStat and AT630 were measured on a spectrophotometer. Average spectra from three independent measurements are shown. Emission peak, λ_{Em}, for ThT (λ_{Em} = 400 nm) was found at 484 nm; ProteoStat emission λ_{Em} = 610 nm (λ_{Ex} = 500 nm); and AT630 emission λ_{Em} = 642 nm (λ_{Ex} = 480 nm). **(B)** Quantum yields and extinction coefficients of ThT, ProteoStat and AT630 were determined as described in **Materials and Methods** and from which brightness were calculated. Alexa647 quantities are taken from the manufacturer (ThermoFisher Scientific), and ThT quantum yield is comparable to that previously observed in water[88].

Figure S2. AT630 and ProteoStat preferentially bind to aggregate structures. **(A)** αS monomers labeled with Alexa647 could not be detected when stained with AT630 or ProteoStat. **(B)** Representative images showing that unstained αS aggregates are not detected without AT630 or ProteoStat staining. Scale bars represent 5 µm.

Figure S3. ProteoStat and AT630 recognize Alexa647-labeled aggregates. **(A)** Schematic representation of aggregate labeling with Alexa647. Recombinant unlabeled and Alexa647-labeled αS monomers were mixed in an optimized ratio of 9:1 prior to aggregation (see **Materials and Methods**). This method ensures that Alexa647-labeled αS are incorporated into all aggregate species (not drawn to scale). **(B)** Reconstruction of an Alexa647-labeled fibril in 3D, assembled according to **A** (see **Video S1**). Scale bar = 500 nm. **(C)** Alexa647-labeled aggregates were stained with either ThT (*top*), ProteoStat (*middle*) or AT630 (*bottom*) and fluorescence emissions from both channels detected simultaneously. Laser power was set at 10 mW for ProteoStat and AT630 and 60 mW for ThT. Scale bars = 2 µm. **(D)** The relationship between fluorescence intensity and size of each aggregate is plotted, color-coded by the fluorophore used for detection (N_{AT630}=136; $N_{ProteoStat}$=206; N_{ThT}=474; $N_{Alexa647}$=213).

Figure S4. Characterization of 2D and 3D SMLM techniques. **(A)** The resolution of the 2D SMLM was calculated by imaging αS monomers labeled with Alexa647 deposited on a plasma cleaned coverslip. The relative positions in the x- and y- directions were calculated from multiple bursts of fluorescence from individual fluorophores, and were plotted in frequency histograms. The FWHM from each histogram represent the possible resolution of the 2D SMLM (N = 17874 fluorescent bursts). **(B)** Similarly, from astigmatic SMLM, the positions in

three dimensions can be plotted in a 3D scatter plot, and are quantified in the x-, y- and z-directions in **(C)** (N = 3741 fluorescent bursts).

Figure S5. Aggregates assembled from αS covalently linked with Alexa647 or Alexa488. (*Left*) Unlabeled and Alexa488- or Alexa647-labeled αS monomers were mixed in a 9:1 ratio and assembled into aggregates. (*Right*) Representative **(A)** Alexa647- and **(B)** Alexa488-linked aggregates detected by diffraction-limited or super-resolved TIRF microscopy. Scale bars = 2 μm.

Figure S6. ProteoStat-stained Aβ and tau aggregates. **(A)** Aggregates assembled from synthetic Aβ42 or **(B)** recombinant tau were stained with AT630 and detected by TIRF microscope, shown as diffraction-limited or super-resolved image (SMLM). Close-ups on typical features of aggregates detected in **A** are shown on the right. Aggregates assembled from recombinant full-length tau (0N4R) carry the P301S mutation. **(D-E)** Similarly, Aβ42 and tau aggregates are imaged with ProteoStat. Scale bars in **A** and **C** represent 1 μm and in **B** and **D** 100 nm.

Figure S7. The cell volume observed using eGFP emission from a typical HEK293A cell expressing proteasome subunit PSMD14-eGFP (green) and compared to the cell volume highlighted using CellMask™ plasma membrane stain. Cells are shown in **(A)** orthogonal views and **(B)** 3D representation of the cell. Scale bar represents 5 μm.

Figure S8. AT630 enables to visualization of aggregates in cells. *(left)* HEK293A cells expressing proteasome subunit PSMD14-eGFP were treated with 1 μM unlabeled αS aggregates and were imaged without staining with AT630. *(right)* HEK293A cells expressing proteasome subunit PSMD14-eGFP were stained with AT630 in the absence of aggregates. Scale bars represent 5 μm, and no significant red signal is detected from either sample.

Figure S9. Typical TIRF images of aliquots taken from αS aggregation reaction. Aggregates were stained with ProteoStat and imaged at **(A)** 0, **(B)** 12 and **(C)** 36 hrs of aggregation. **(D)** An image of sonicated sample from **C**. All images were set to the same intensity range. Scale bars = 5 μm.

Figure S10. Brain soak aggregate extraction from post-mortem PD and DLB donor brain tissues. **(A)** Cartoon representation of the experimental workflow for extraction of aggregates from donor brain tissues. **(B)** Aggregates extracted from three PD and three DLB samples were separated on a 4-12% SDS-PAGE and stained with Coomassie (see **Table S1**). Ladder (L) for molecular weights is indicated. **(C)** Samples from **B** were transferred to a PVDF membrane and stained with an anti-αS antibody (MJRF1). **(D)** The brain soak samples were separated on a 4-16% NativePAGE and stained with Coomassie.

Figure S11. The relationship between cytotoxicity and $aggregate_{450nm}$ concentrations of PD1, PD2 and PD3 (see **Table 1**), show a linear relationship between 0-1 µM range (purple dashed line), and compared to the linear fit (red solid line) described in **Figure 4F** for the recombinant αS samples.

Figure S12. Cytotoxicity from LDH assay is not altered by prolonged incubation and independent of fibril concentration. **(A)** The cytotoxicity levels of the PD and DLB samples at 4 hrs and 24 hrs (black and red bars, respectively) show no significant (n.s.) difference in cytotoxicity between samples incubated for the two different times. Mean values and error bars represent standard deviation of three independent measurements. **(B)** (*left*) The relationship between cytotoxicity and the composition of heterogeneous samples. PD derived aggregates were mixed with αS fibrils keeping the overall monomer-equivalent concentration at 5 µM, and were used in an LDH assay to characterize each samples cytotoxicity. (*right*) LDH assays were also performed on cells incubated with increasing concentrations of fibrils alone (1, 5, 10 µM) as a comparison to the cytotoxicity levels in **B**.

Figure S13. Comparison between azimuthal or “spinning”- and conventional TIRF. (**A**) Four images taken from 0°, 90°, 180° and 270° azimuthal angles of the same field-of-view of a 10 µm mouse brain tissue section under HILO imaging. (**B**) Average intensity from the four images from **A**. Scale bars = 10 µm. **(C)** Fluorescence intensity profile plotted from the dashed line in **B** for (*top*) each of the four images in **A** and (*bottom*) the averaged image in **B**, showing background suppression with spinning TIRF.

Video S1. Alexa647-labeled fibril super-resolved in 3D. See also **Figure S3B**.

Video S2. SMLM of Alexa647-αS aggregates. See also **Figure S5**.

Video S3. SMLM of ProteoStat-stained unlabeled αS aggregates. See also **Figure S6D**.

Video S4. SMLM of AT630-stained unlabeled αS aggregates. See also **Figure S6D**.

Video S5. Colocalization of proteasome foci and Alexa647-αS aggregates super-resolved in 3D. See also **Figure 3C**.

Video S6. AT630-stained small aggregate species super-resolved in 3D. See also **Figure 4D**.

Video S7. AT630-stained aggregates in $App^{NL\text{-}G\text{-}F}$ mouse brain tissues super-resolved in 3D. See also **Figure 6B**.

Table S1. Details of post-mortem brain samples used.

Case	Age	Sex	PMDelay (hrs)	Concentration (mg/mL)	PathDiagnosis
PD1	76	M	66	0.48	Parkinson’s Disease
PD2	85	F	9	0.44	Parkinson’s Disease
PD3	69	F	34	0.84	Parkinson’s Disease
DLB1	80	F	40	0.44	Dementia with Lewy bodies
DLB2	71	M	19	0.60	Dementia with Lewy bodies
DLB3	66	M	53	0.75	Dementia with Lewy bodies

Figure S1

A

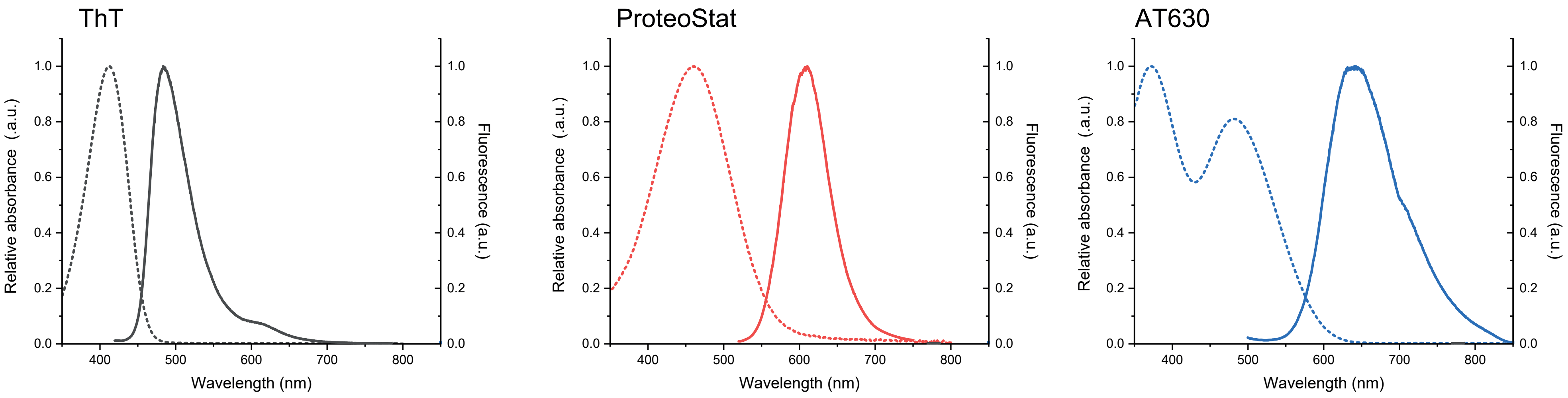

B

Fluorophore	Quantum Yield	Extinction Coefficient ($M^{-1}cm^{-1}$)	Brightness
ThT	0.0030	27800	41.3
ProteoStat	0.212	81084	17189
AT630	0.245	14049	3442
Alexa647	0.33	270000	89100

Figure S2

A

Alexa647-labeled αS monomers				
excited at 638 nm	+	-	-	-
excited at 561 nm	-	+	+	+
AT630	-	-	+	-
ProteoStat	-	-	-	+

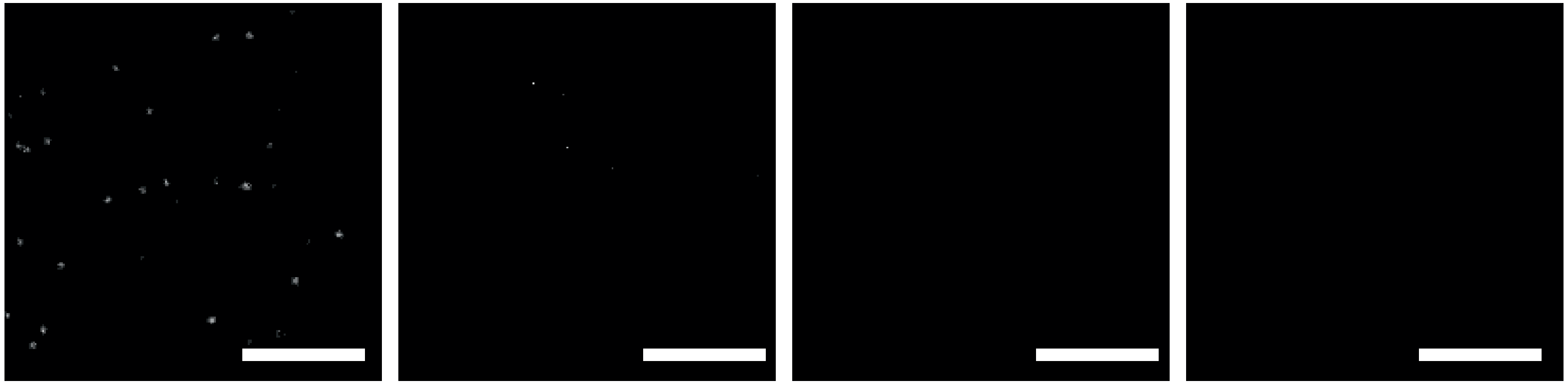

B

Unlabeled aggregates	+	+	+
AT630	-	+	-
ProteoStat	-	-	+

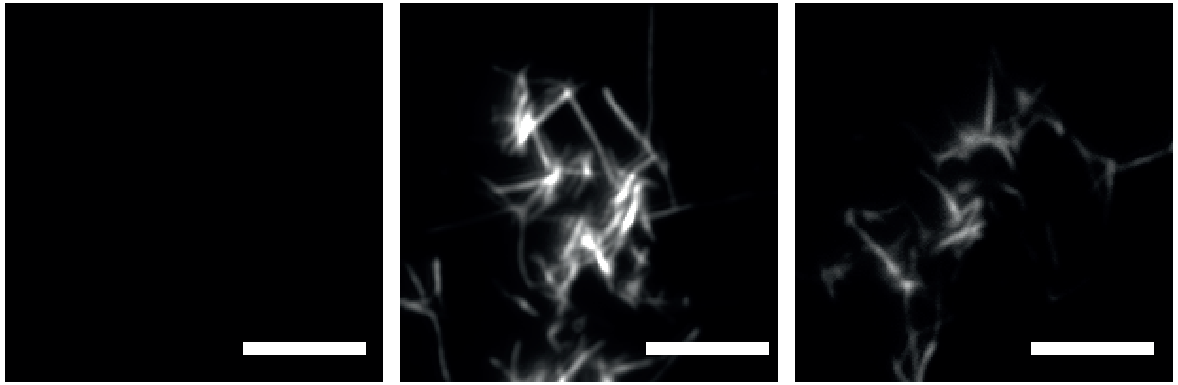

Figure S3

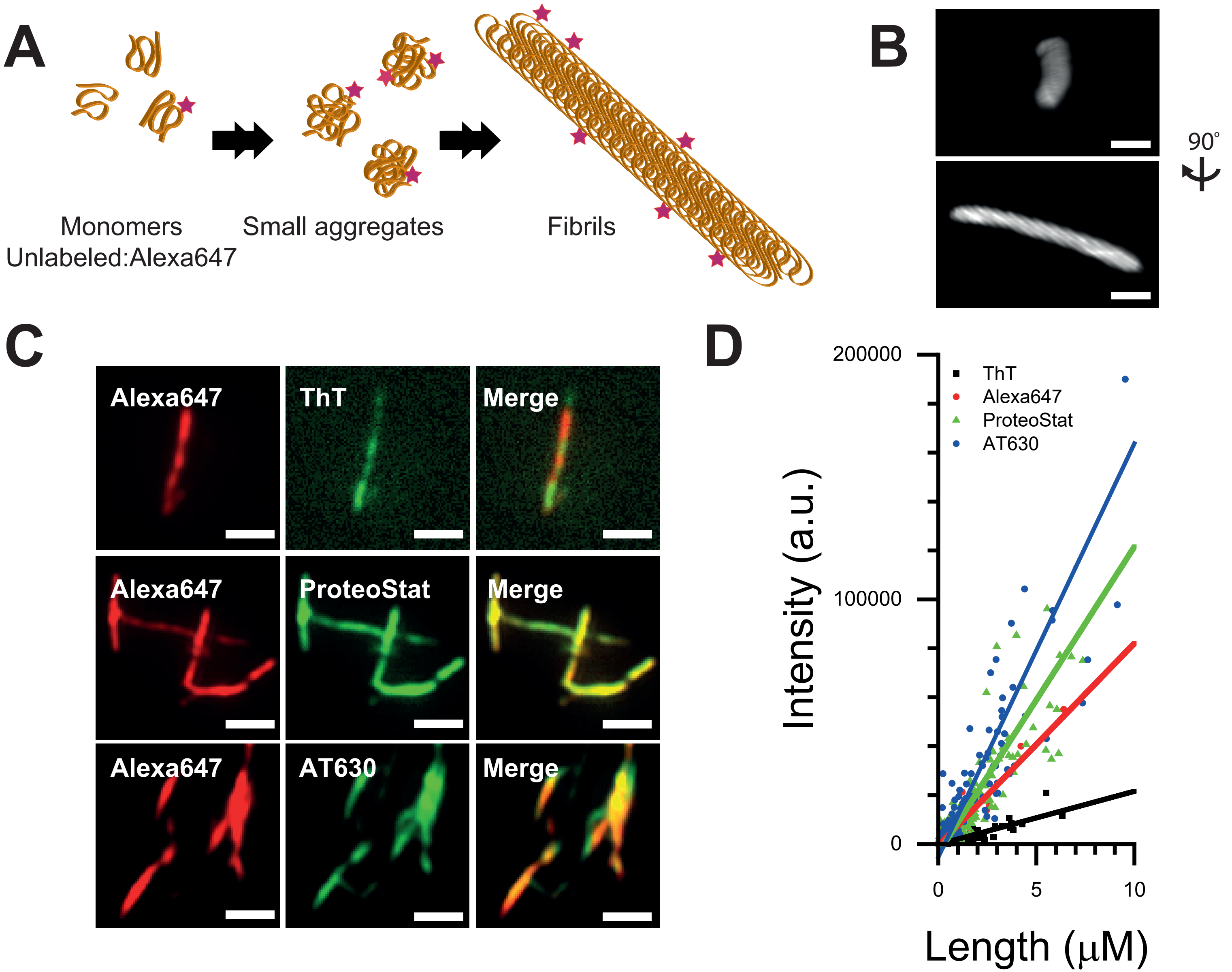
A
Monomers
Unlabeled:Alexa647
Small aggregates
Fibrils
B
90°
C
Alexa647
ThT
Merge
Alexa647
ProteoStat
Merge
Alexa647
AT630
Merge
D
200000
100000
0
ThT
Alexa647
ProteoStat
AT630
Intensity (a.u.)
0
5
10
Length (μM)

Figure S4

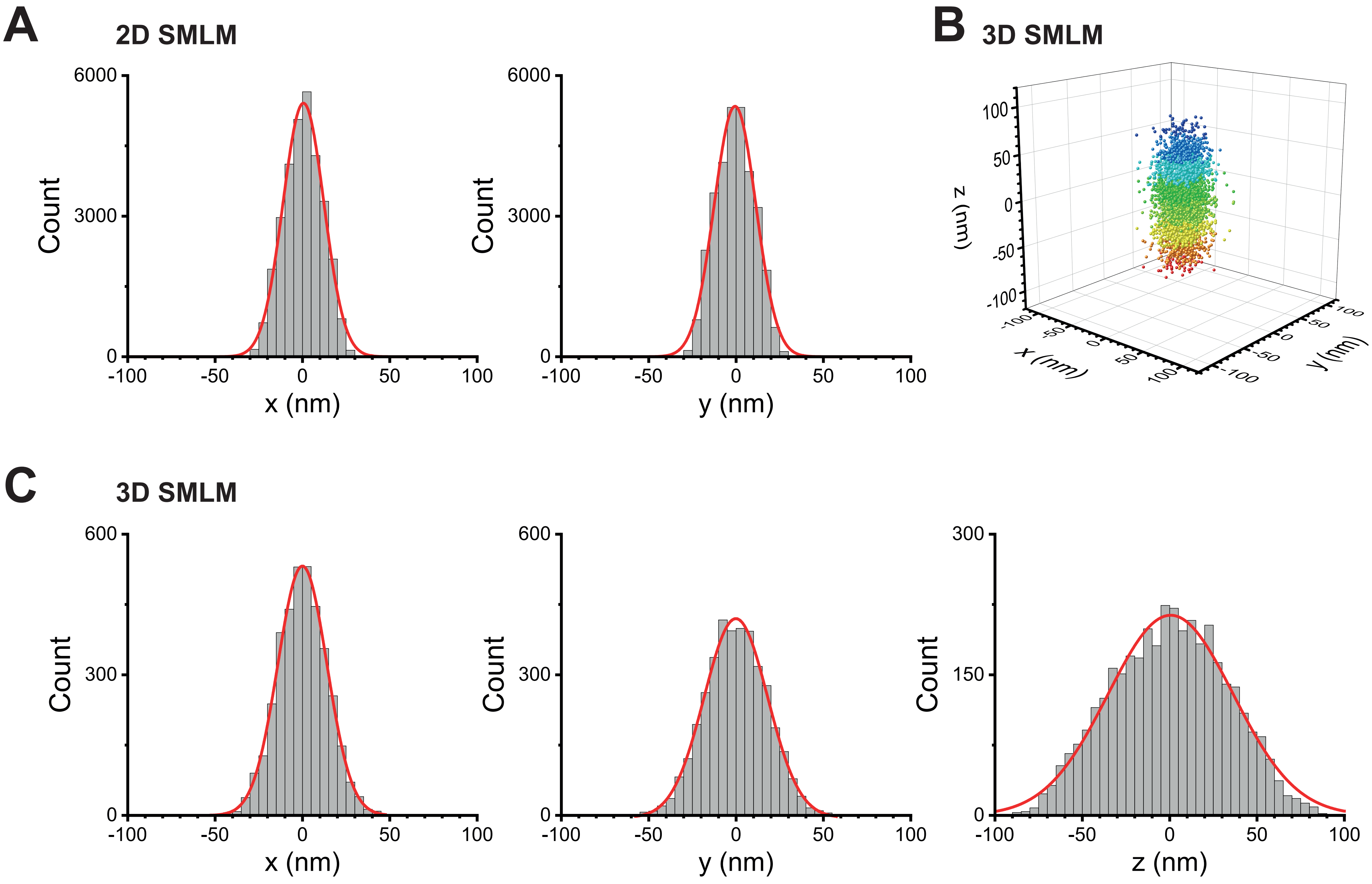
A
2D SMLM
Count
6000
3000
0
-100
-50
0
50
100
x (nm)
Count
6000
3000
0
-100
-50
0
50
100
y (nm)
B
3D SMLM
z (nm)
100
50
0
-50
-100
x (nm)
y (nm)
C
3D SMLM
Count
600
300
0
x (nm)
Count
600
300
0
y (nm)
Count
300
150
0
z (nm)

Figure S5

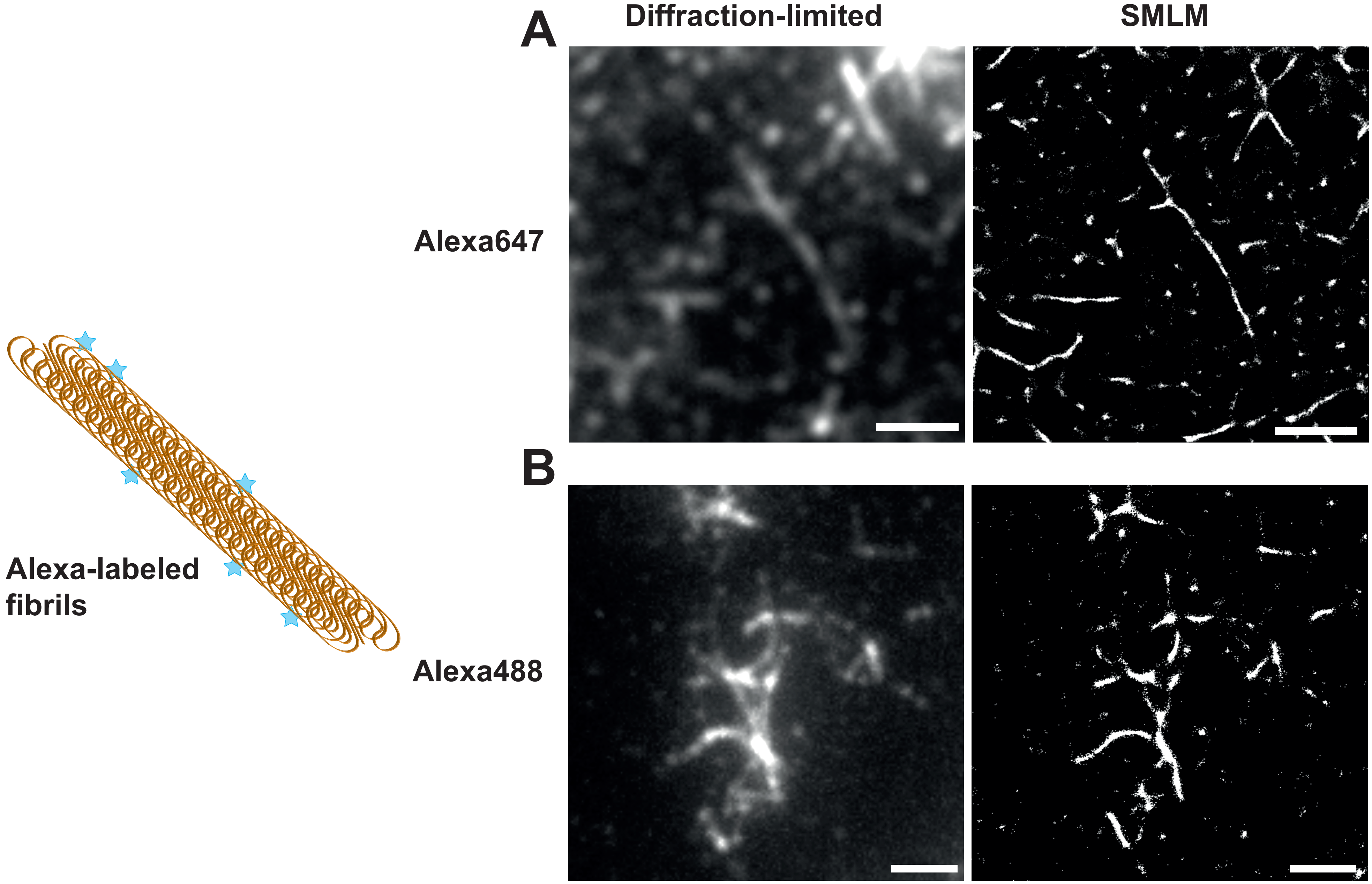
A
Diffraction-limited
SMLM
Alexa647
B
Alexa488
Alexa-labeled
fibrils

Figure S6

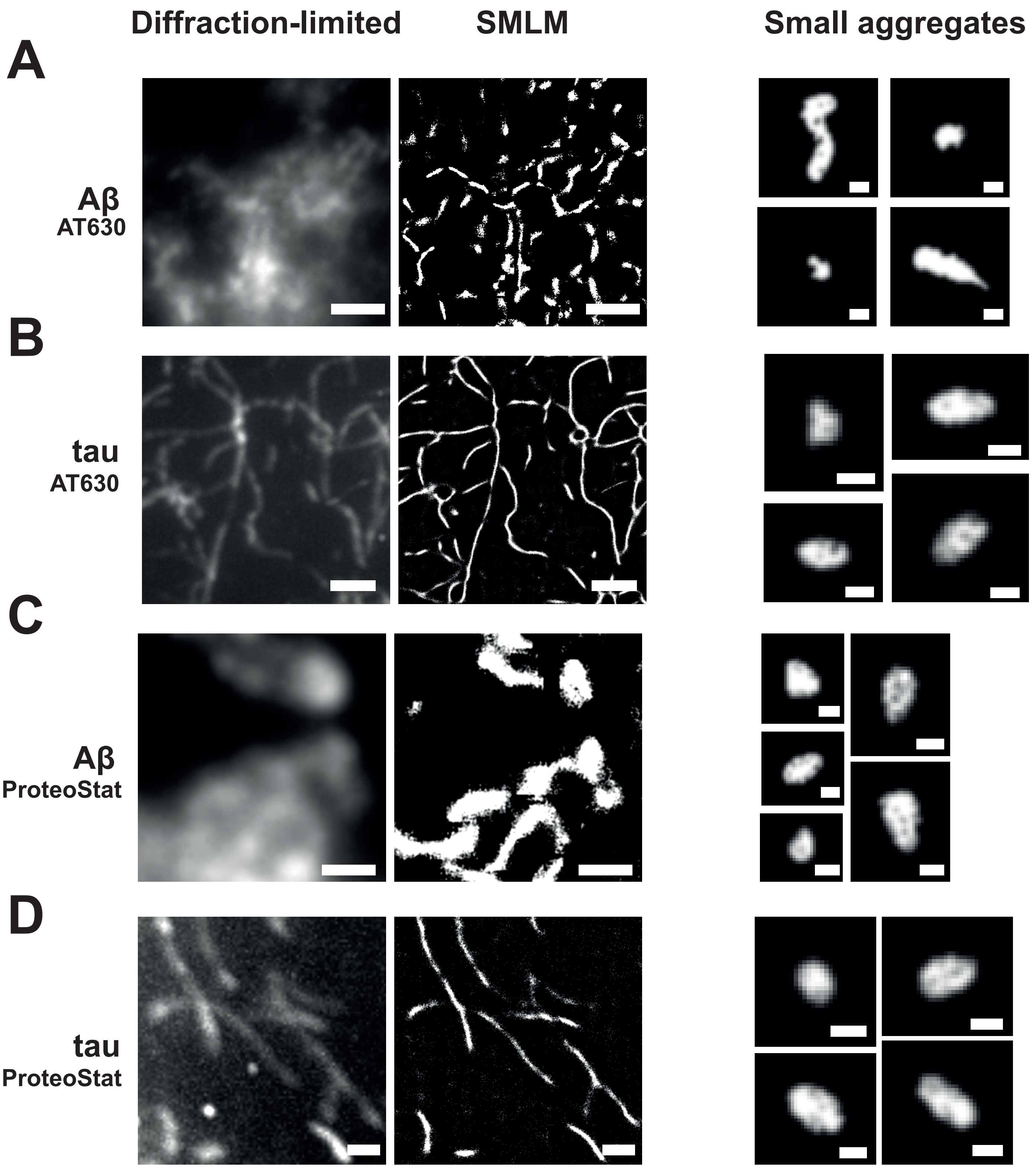

Diffraction-limited
SMLM
Small aggregates
A
Aβ
AT630
B
tau
AT630
C
Aβ
ProteoStat
D
tau
ProteoStat

Figure S7

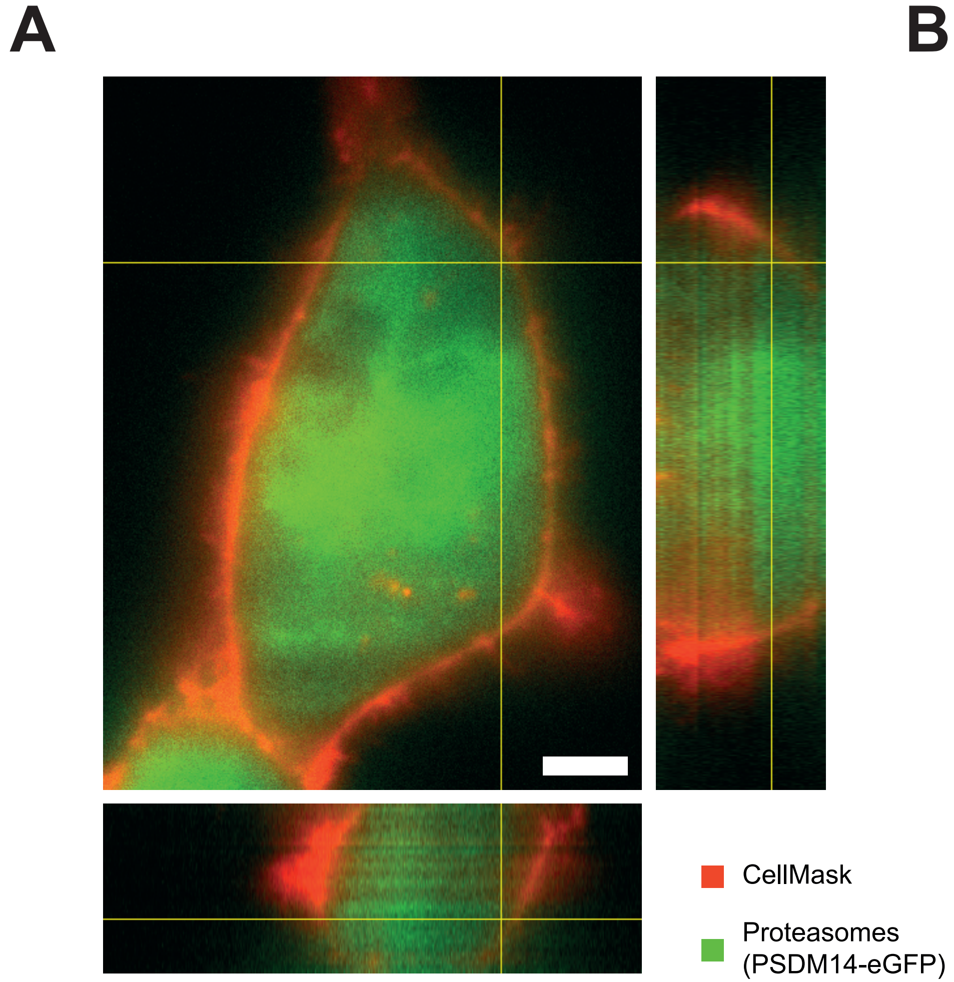

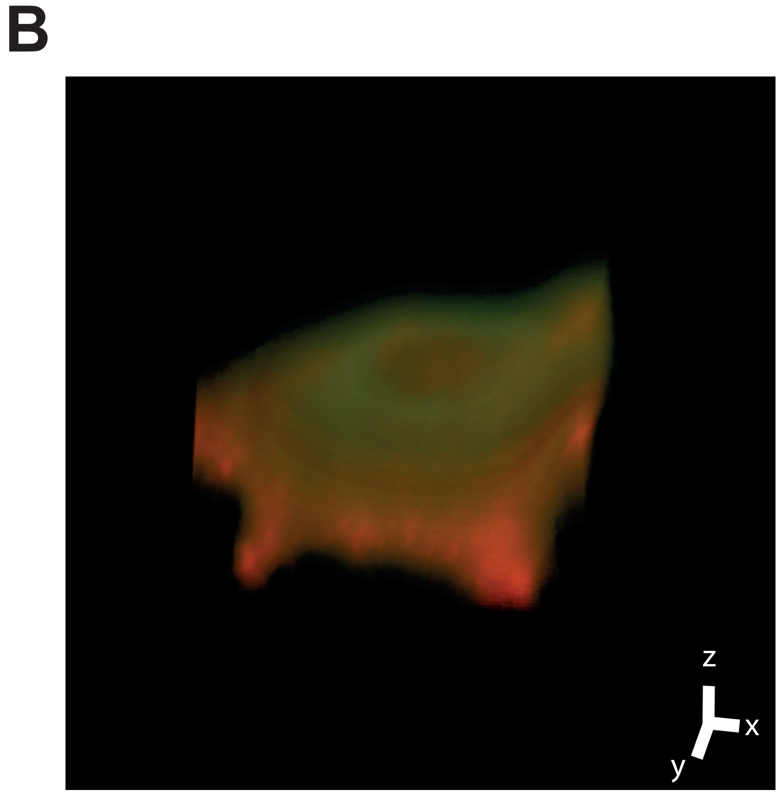

Figure S8

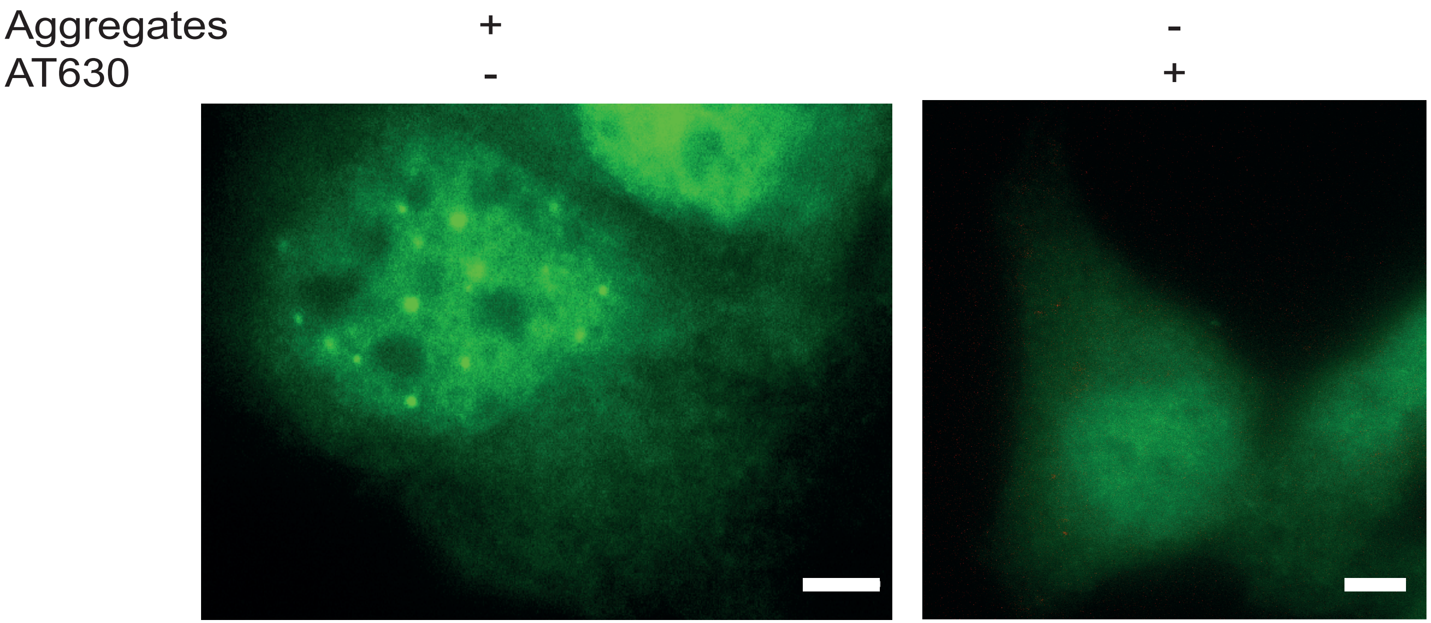

Figure S9

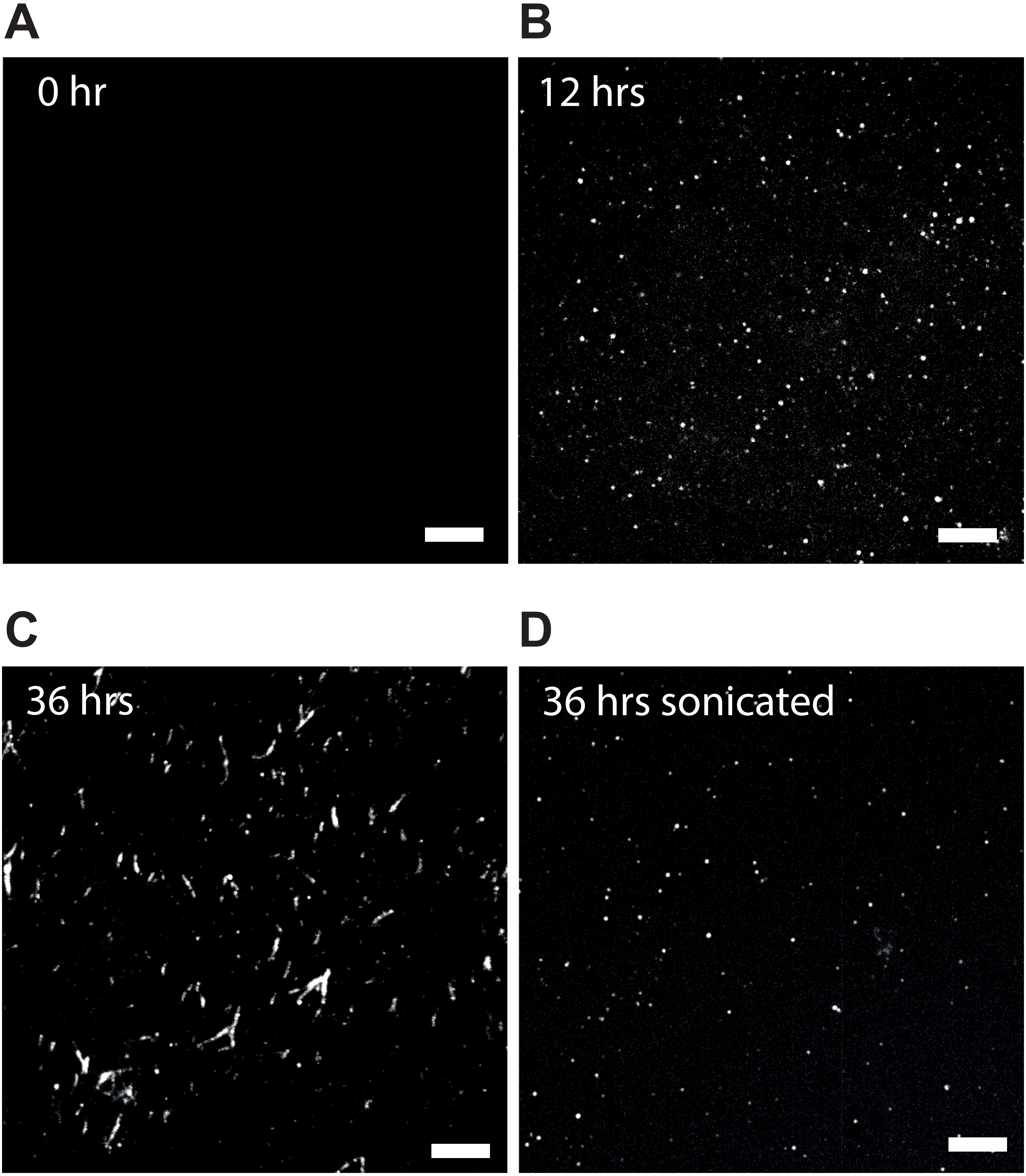

Figure S10

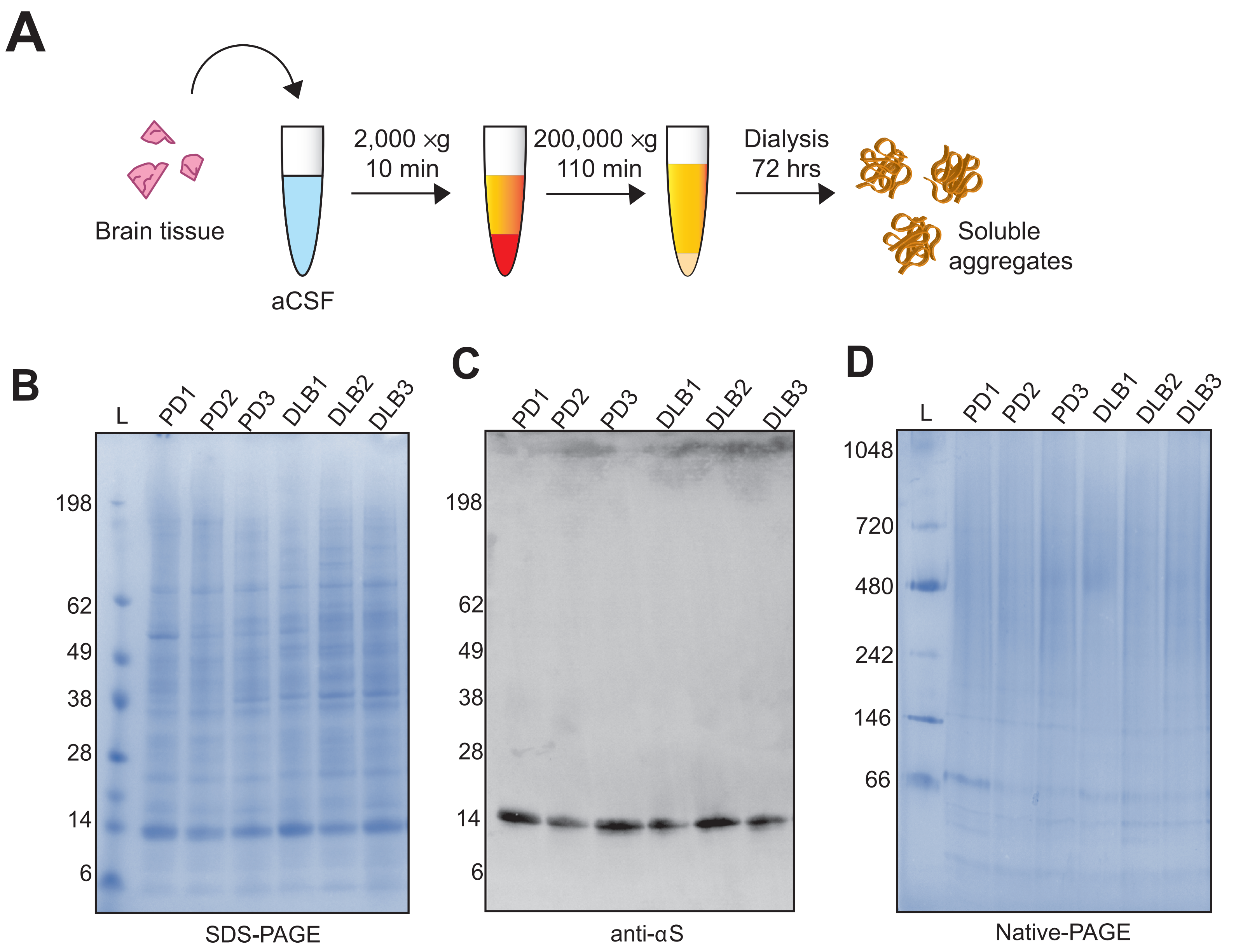
A
Brain tissue
aCSF
2,000 ×g
10 min
200,000 ×g
110 min
Dialysis
72 hrs
Soluble
aggregates
B
L
PD1
PD2
PD3
DLB1
DLB2
DLB3
198
62
49
38
28
14
6
SDS-PAGE
C
PD1
PD2
PD3
DLB1
DLB2
DLB3
198
62
49
38
28
14
6
anti-αS
D
L
PD1
PD2
PD3
DLB1
DLB2
DLB3
1048
720
480
242
146
66
Native-PAGE

Figure S11

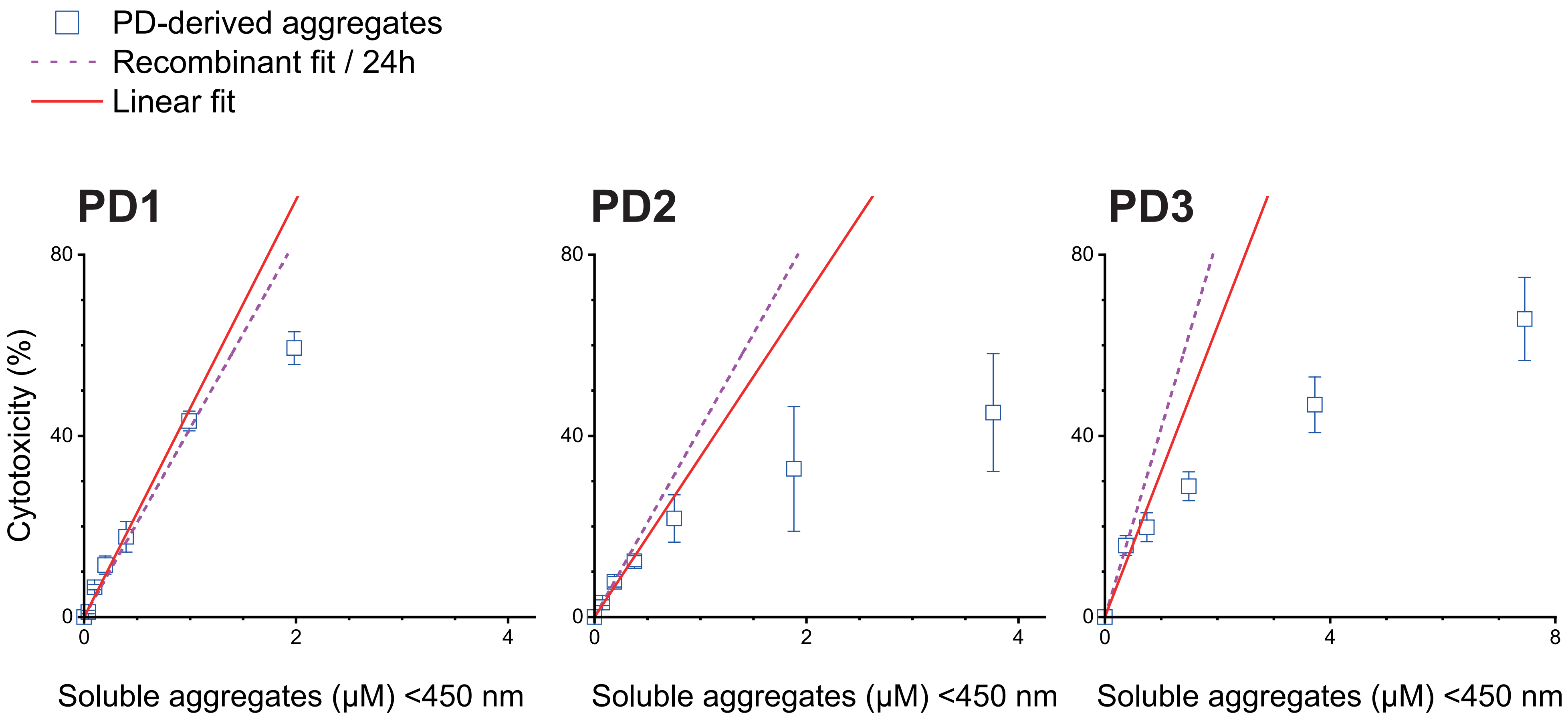
PD-derived aggregates
Recombinant fit / 24h
Linear fit
PD1
PD2
PD3
Cytotoxicity (%)
80
40
0
0
2
4
8
Soluble aggregates (μM) <450 nm

Figure S12

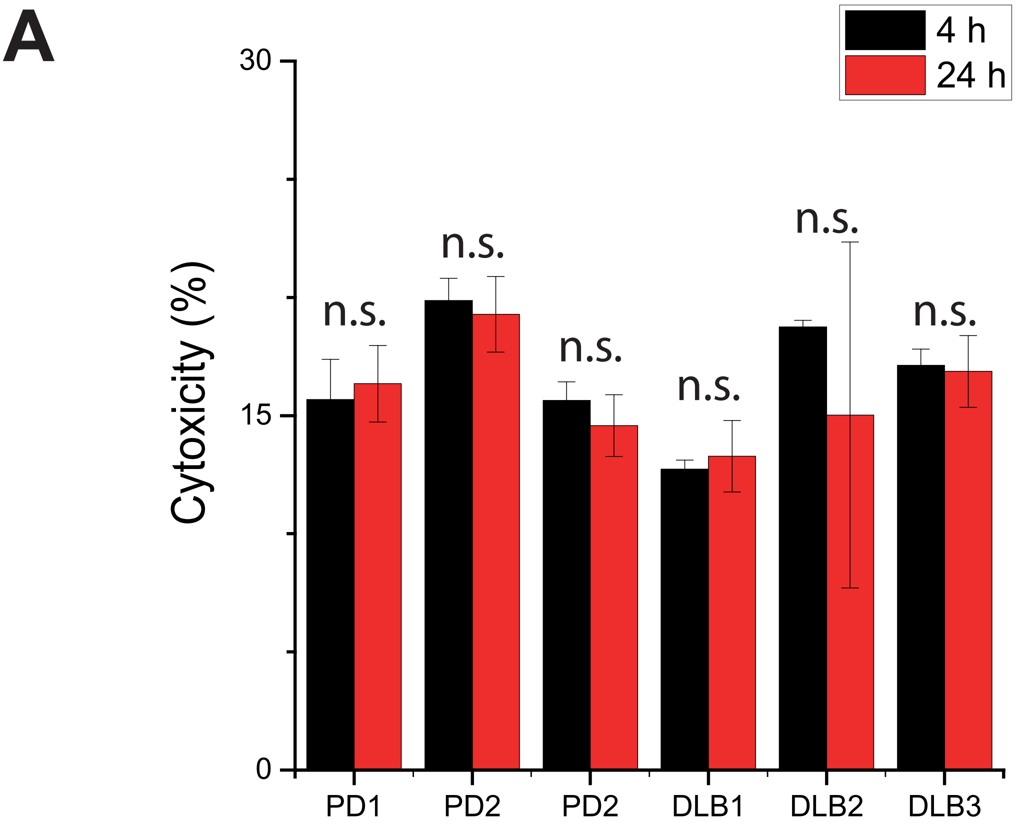

A
4 h
24 h
30
15
0
Cytoxicity (%)
n.s.
n.s.
n.s.
n.s.
n.s.
n.s.
PD1
PD2
PD2
DLB1
DLB2
DLB3

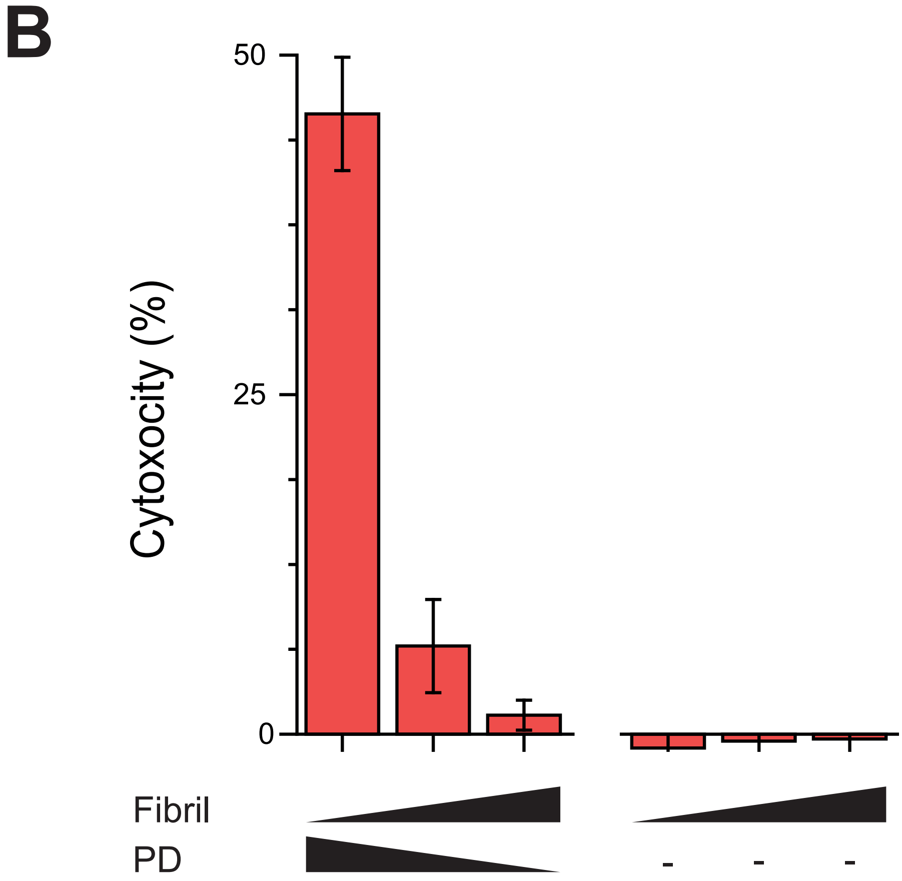

B
50
25
0
Cytoxocity (%)
Fibril
PD
-
-
-

Figure S13

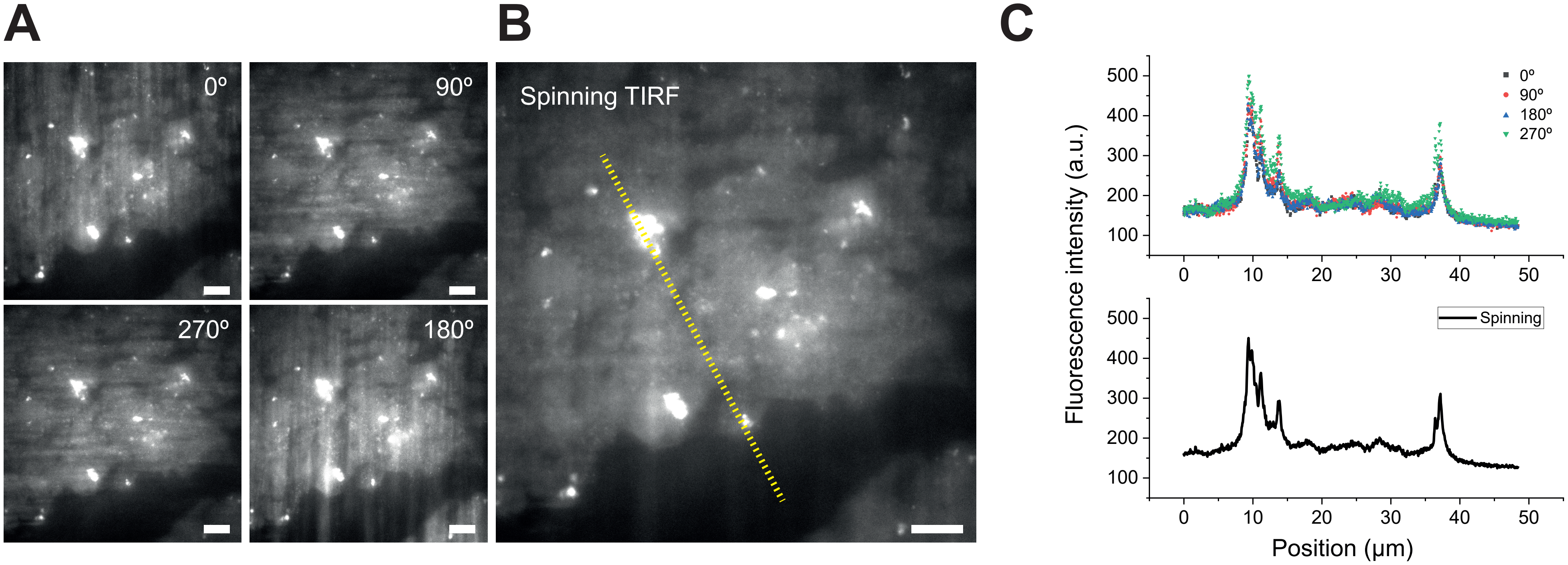
A
0º
90º
270º
180º
B
Spinning TIRF
C
Fluorescence intensity (a.u.)
0º
90º
180º
270º
Spinning
Position (µm)
0
10
20
30
40
50
100
200
300
400
500
